# Modeling the Effect of Defects and Disorder in Amorphous Metal–Organic Frameworks

**DOI:** 10.1021/acs.chemmater.2c01528

**Published:** 2022-10-13

**Authors:** Irene Bechis, Adam F. Sapnik, Andrew Tarzia, Emma H. Wolpert, Matthew A. Addicoat, David A. Keen, Thomas D. Bennett, Kim E. Jelfs

**Affiliations:** † Department of Chemistry, Imperial College London, Molecular Sciences Research Hub, White City Campus, London W12 0BZ, U.K.; ‡ Department of Materials Science and Metallurgy, University of Cambridge, Cambridge CB3 0FS, U.K.; § School of Science and Technology, Nottingham Trent University, Clifton Lane, Nottingham NG11 8NS, U.K.; ∥ ISIS Neutron and Muon Facility, Rutherford Appleton Laboratory, Harwell Campus, Didcot, Oxfordshire OX11 0QX, U.K.

## Abstract

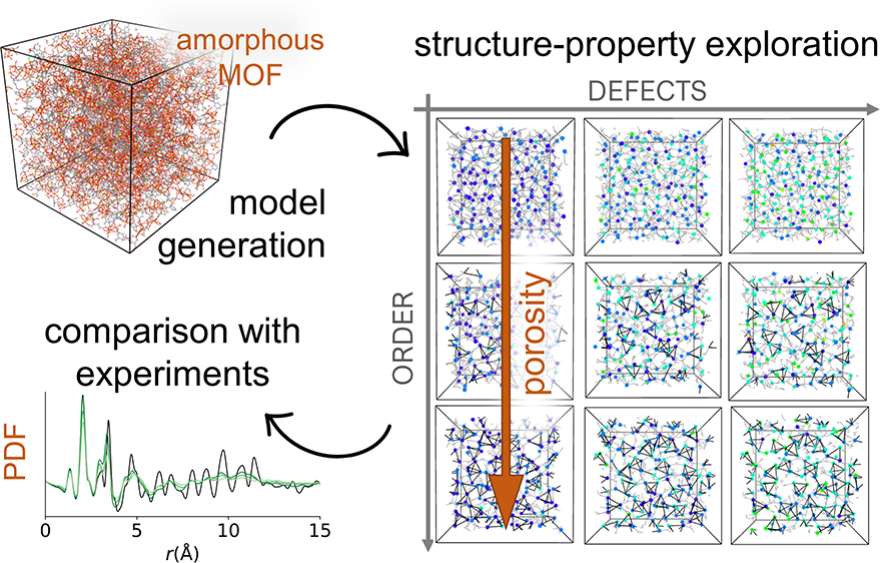

Amorphous metal–organic frameworks (*a*MOFs) are a class of disordered framework materials with a defined local order given by the connectivity between inorganic nodes and organic linkers, but absent long-range order. The rational development of function for *a*MOFs is hindered by our limited understanding of the underlying structure–property relationships in these systems, a consequence of the absence of long-range order, which makes experimental characterization particularly challenging. Here, we use a versatile modeling approach to generate *in silico* structural models for an *a*MOF based on Fe trimers and 1,3,5-benzenetricarboxylate (BTC) linkers, Fe-BTC. We build a phase space for this material that includes nine amorphous phases with different degrees of defects and local order. These models are analyzed through a combination of structural analysis, pore analysis, and pair distribution functions. Therefore, we are able to systematically explore the effects of the variation of each of these features, both in isolation and combined, for a disordered MOF system, something that would not be possible through experiment alone. We find that the degree of local order has a greater impact on structure and properties than the degree of defects. The approach presented here is versatile and allows for the study of different structural features and MOF chemistries, enabling the derivation of design rules for the rational development of *a*MOFs.

## Introduction

Metal–organic frameworks (MOFs) are versatile materials constructed from inorganic nodes and organic linkers.^1^ As the combination of nodes and linkers forms an open and porous framework, MOFs can show high surface areas and pore volumes, making them promising candidates for applications including gas separations, storage, and catalysis.^2^ Due to the almost endless possible combinations of their building blocks and the control over their organization in 3-dimensional space arising from strong directional bonding, the properties of these materials can potentially be tuned for specific functionalities.^3^ Though MOFs are typically regarded as having perfectly ordered structures, it has become increasingly clear that many deviate from perfect crystallinity in the presence of disorder.^4−6^

Disorder can be introduced into MOFs in a variety of ways, and one of the most studied is that of defects. Defects are small deviations from the perfect crystalline framework, such as a missing linker (ML) or a missing node (MN), which break the regular periodic arrangement of atoms, and are ubiquitous in MOFs.^7,8^ An increasing number of studies have been focusing on defect engineering in MOFs, as the controlled introduction of defects allows for fine-tuning the materials’ physicochemical properties, such as porosity, catalytic activity, and mechanical, optical, or electromagnetic properties.^9,10^

Defects however are not the only form of disorder that can exist in MOFs. Amorphous MOFs (*a*MOFs), which include MOF glasses,^11^ liquids,^12^ and some gels,^13^ are materials that show topological disorder, as they maintain a well-established local order defined by the metal–linker connectivity, but lack long-range order.^14−16^*a*MOFs can be formed by direct synthesis if the reaction between the MOF precursors avoids crystallization and leads to an amorphous gel,^13^ or by applying pressure,^17−19^ temperature,^20−22^ or mechanical stress^23−26^ to the parent crystalline framework.

Typically, the loss of order causes a reduction in void space in the system compared to crystalline MOFs. Only a few examples of *a*MOFs are reported to maintain accessible porosity to guests like CO_2_ after melt-quenching.^27−29^ However, increasing the porosity of a system is rarely the sole driver of potential function in a porous material. Other drivers include the diffusion of small guests such as ions in more dense structures,^30^ the absence of grain boundaries, and the resulting possibility to shape the material into useful bulk morphologies,^13^ making *a*MOFs particularly promising from an application stand point. It remains an open question as to whether it is inevitable that topological disorder in an *a*MOF must result in a reduction of porosity relative to the crystalline, ordered framework or whether exceptions to this rule exist. It is also unclear to what degree tuning of porosity for a given function, for example, pore size control for selective guest diffusion, is possible for disordered MOFs. To help answer these questions, design principles that allow for the control of porosity in these systems are much needed.

Developments within the field of *a*MOFs are hindered by the increased difficulty in their structural characterization compared to their crystalline counterparts. The absence of long-range periodicity means that *a*MOFs are harder to characterize using conventional diffraction techniques. Advanced diffraction techniques, such as total scattering, that analyze both Bragg and diffuse scattering signals have proved very useful in gaining information on the local structure of *a*MOFs.^20,31,32^ In particular, we have previously shown how the pair distribution function (PDF) can be essential in characterizing *a*MOFs, as it provides real-space information regarding the local structure of the system.^33^

For porous crystalline materials, structural determination using X-ray diffraction has enabled the collection of large databases of structures such as the Cambridge Structural Database,^34^ the database of zeolite structures by the International Zeolite Association,^35^ or the Computation-Ready Experimental Metal–Organic Framework^36,37^ database. Databases of materials are valuable in opening the door to advances in materials discovery through the application of data-science tools and machine learning. By contrast, the field of amorphous porous materials is far behind, having only recently seen the publication of the first structural database of amorphous microporous materials, including 205 structures of carbons, polymers, kerogens, and one example of an *a*MOF.^38^ The absence of a unique crystallographic unit cell makes modeling amorphous materials more challenging and limits the predictive capabilities of computational researchers in the field of *a*MOFs.^39^

To overcome such challenges, previous studies on modeling *a*MOFs have used reverse mapping approaches, where models of simpler amorphous inorganic materials are transformed to possess the correct chemistry of a MOF and then in some cases refined against experimental data (e.g., using reverse Monte Carlo modeling).^20,40−42^ Alternatively, direct simulations of the amorphization procedure from a crystalline structure can be performed using molecular dynamics (MD). These approaches must include a description of the bond-breaking and -forming events present during amorphization, which has been achieved in *a*MOFs using the reactive force field ReaxFF in classical simulations or, alternatively, using *ab initio* MD simulations.^12,43−46^ The first approach is limited in the number of MOF chemistries the force field covers, while the second, although very useful for providing mechanistic insights, is too computationally expensive to be used as a routine method to produce structures of *a*MOFs, given the size needed to reproduce the amorphous system.

To overcome the issues mentioned above, we have recently presented an approach for constructing large structural models for *a*MOFs without requiring any experimental data as the input.^30,33^ The approach, which is adapted from the field of polymer modeling, allows for predictive modeling of *a*MOFs and is applicable to most MOF chemistries when coupled with a generic MOF force field.^47,48^ Structures generated in this way can be directly used for property evaluation or as a starting point for further analysis and refinement. Our approach, based on a customizable polymerization algorithm (Polymatic)^49^ that creates bonds between nearby building blocks of the *a*MOF, allows for the exploration of the amorphous phase space, removing the need for a reactive force field that describes bond breaking/formation. The approach presented is versatile, not only in the diversity of chemistries that it can cover, but also in its ability to go beyond known structures, which we use here to explore the effects of different degrees of defects and disorder on the structure of an *a*MOF with fixed chemical composition.

**Figure 1:**
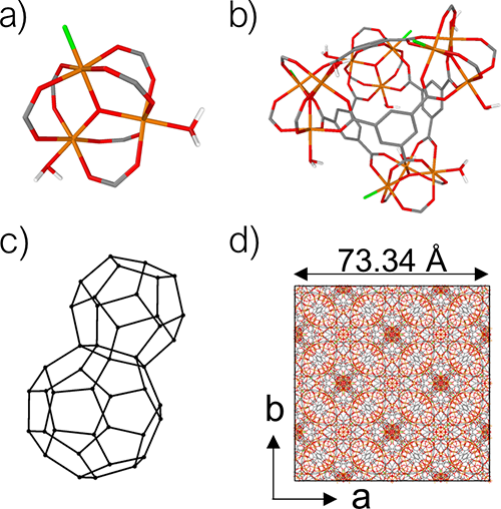
(a) Oxo-centered iron trimer. (b) Assembly of four trimers and four BTC linkers in a tetrahedron (hydrogen atoms in the linker removed for clarity). (c) Schematic of the **mtn** topology, where the centroids of the tetrahedra (black dots) occupy the nodes of the network. (d) Unit cell of MIL-100 (Fe). Carbon is shown in gray, oxygen in red, iron in orange, fluorine in green, and hydrogen in white.

We focus on the disordered MOF Fe-BTC, also known under the commercial name of Basolite F300. X-ray adsorption studies^50^ and PDF data^33,51^ have shown that Fe-BTC has the same local structure as the highly porous, crystalline MOF, MIL-100(Fe), with the same chemical composition [Fe^III^_3_O(H_2_O)_2_F·{C_6_H_3_(CO_2_)_3_}_2_].^52^ Both materials exhibit oxo-centered trimers of iron(III) octahedra, in which each iron atom coordinates four 1,3,5-benzenetricarboxylate (BTC) linkers and a water molecule or F^–^ anion in terminal position (Figure 1a). In MIL-100(Fe), these trimers further assemble into tetrahedral structures, each containing four trimers and four BTC linkers (Figure 1b), which then assemble into an **mtn** topology (Figure 1c,d). Fe-BTC, on the other hand, is a composite material, made up of nanocrystals embedded in an amorphous matrix. By a combination of total scattering measurements and simulations, we recently showed that the amorphous matrix of Fe-BTC incorporates a certain degree of these tetrahedra too.^33^ The trimer substructure defines the short-range local order (SRO) of the material, while the tetrahedron is characteristic of its medium-range local order (MRO).

Fe-BTC has been abundantly studied as a catalyst for oxidation reactions,^53^ because its structure includes both Lewis sites (the terminal positions on iron(III) atoms in the trimer) and Brønsted acid sites that are ascribed to defective terminal carboxylic groups of protonated linkers that disrupt the network.^50,54,55^ Fe-BTC has been reported to have a broad range of structural diversity, resulting in a family of materials showing different properties depending on the synthetic route adopted. As an example, the BET surface area of Basolite F300, likely produced through an electrochemical method,^56^ is reported as lying between 1300 and 1600 m^2^ g^–1^, but several reports found different values, ranging from 685 to 1055 m^2^ g^–1^.^51,54,55,57−60^ Similarly, studies on Fe-BTC obtained by a sol–gel route report BET surface area values between 0 and 1618 m^2^ g^–1^,^33,55,58−62^ while the amorphous phase obtained by ball-milling MIL-100(Fe), *a_m_*MIL-100, is non-porous.^63^ All these reported phases share the same composition and local structure but have different properties in terms of porosity. For this reason, we selected Fe-BTC as a model system to test the versatility of our approach in exploring amorphous phases with different structural features.

Here, we built nine amorphous phases with Fe-BTC composition but different degrees of local order and defects. We characterized these phases to rationalize the individual and combined effects of disorder and defects on the models’ properties, in particular, their porosity. Finally, we compared our models to experimental phases and used our approach to probe the mechanism of framework collapse during ball-milling. This work is a step forward toward establishing design principles for controlling porosity in *a*MOFs and shows the potential of computational modeling in accelerating the discovery of new phases in this family of materials.

## Methods

**Figure 2:**
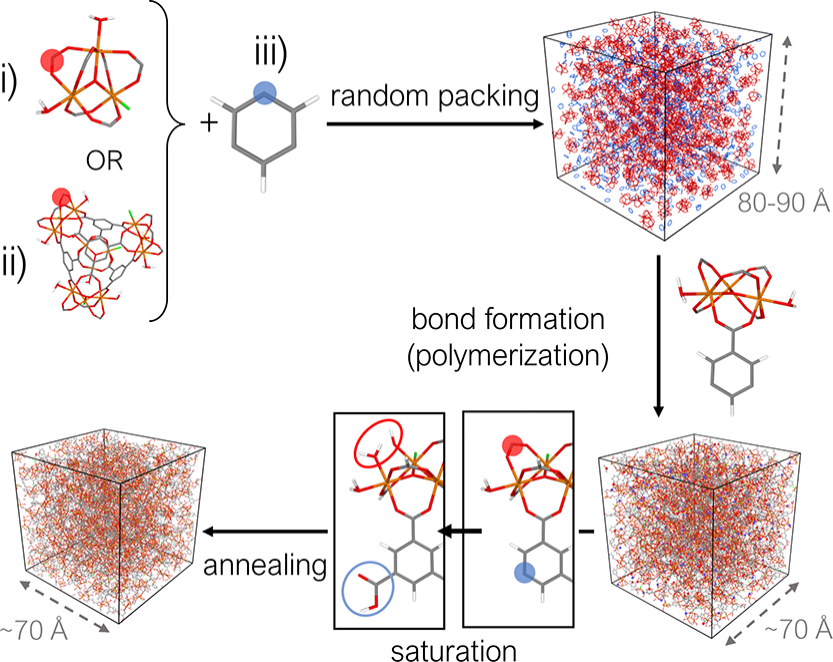
Schematic of the workflow used to build the structure of the amorphous models. After packing the building blocks of the system in a periodic box, bonds are formed by the Polymatic algorithm between reactive sites within a 5 Å cutoff. Each trimer/tetrahedron/linker has 6, 12, and 3 reactive sites, respectively. The figure highlights one reactive site for each building block, with filled red circles for the trimer (i) and tetrahedron (ii) and a filled blue circle for the linker (iii). The sites that remain unreacted after the polymerization are subsequently saturated with a −COOH group (empty blue circle) on the BTC linker and OH/H_2_O groups (empty red circles) on the trimer/tetrahedron. Finally, the structure is annealed through a 21-step MD protocol.

In this work, we study nine amorphous phases at different degrees of disorder and defects, three of which have already been published in a previous study^33^ on the structure of an Fe-BTC material obtained by a sol–gel approach. The amorphous models were built using the polymerization algorithm Polymatic,^49^ which we showed to describe the structure of amorphous microporous polymers well,^64,65^ and that we have previously used to build the structure of *a*ZIFs.^30^ Our workflow is outlined in Figure 2 and includes four steps: random packing of constituent building blocks, bond formation (polymerization), saturation, and annealing.

Initially, the building blocks of the amorphous structure were randomly packed at low density (around 0.6–0.8 g cm^–3^) in a periodic cubic box with a cell length of 80–90 Å (Table S3). The initial low density is necessary to ensure building blocks mobility in the following steps of the workflow. We have previously found the structure at the end of the process to be independent of the starting density. We used either the trimer (Figure 2i), or the tetrahedron (Figure 2ii), or a mixture of the two in combination with the BTC linker (Figure 2iii) as starting building blocks for our amorphous structures. These give what we will refer to as SRO, MRO, and MIX (as it includes a mixture of trimers and tetrahedra) phases, respectively. After the initial random packing, bonds are created between randomly selected reactive sites on opposite building blocks that fall within a defined cutoff distance (5 Å). With our computational workflow, we do not aim to model the experimental bond formation process, but we aim to efficiently generate representative models with the correct node-linker connectivity. With this goal in mind, for the sake of computational simplicity, the bond formed in the polymerization step is not the coordination bond between the oxygen on the BTC linker and the iron on the trimer, but the bond between the carboxylate carbon (filled red circle in Figure 2i,ii) and the adjacent aromatic carbon on the BTC linker (filled blue circle in Figure 2iii). The bond formation is helped by intermediate MD cycles, which allow for the relaxation of the structure and bring additional reactive site pairs within the cutoff distance of each other for potential selection for new bond formation.

At the end of the polymerization stage, when no additional reactive sites can be found within the cutoff distance, the remaining unreacted sites are saturated with OH/H_2_O groups on the two iron atoms left uncoordinated on the trimer and with a carboxylic acid functionality on the unreacted BTC linker (empty circles in red and blue in Figure 2, respectively). The saturation step corresponds to the overall introduction of two additional oxygens and four additional hydrogens to the model for each missed bond and represents the insertion of a defect in the structure. Hence, by controlling the degree of polymerization, we can control the number of missed bonds and consequently the number of defects introduced in the structure. We note that, as a result of our choice to form carbon–carbon bonds instead of the iron–oxygen coordination bonds, it would not be possible to have linkers binding in a monodentate fashion, a defect type that has been suggested by studies on MIL-100(Fe).^66^ In our models, each carboxylate of the BTC linker is either coordinated in a bidentate fashion or uncoordinated (in the carboxylic acid form).

In the final stage, the structure is annealed using an established protocol^33,67^ that includes 21 MD steps and reaches high temperatures (1000 K) and high pressures (5 × 10^4^ bar) to allow the system to relax and increase its density. Further details on the amorphous structure generation workflow can be found in Section S1.2. Only the structures obtained after annealing, which have a more realistic density, were used for characterization. An assessment of the effect of model size, reported in Section S1.2.5, shows that the selected box size allows for a good exploration of all the phases at different disorder levels and represents a good middle ground between computational efficiency and accuracy.

The whole procedure was performed in the gas phase and the structures were described using the UFF4MOF force field,^47,48^ which we validated for this system (details in Section S1.1). All the simulations were performed in LAMMPS,^68^ and the porosity of the resulting structures was analyzed using Zeo++, which performs a geometry-based analysis of the voids inside the model.^69^ This analysis includes the calculation of the pore size distribution (PSD), surface area and volume values, and the diameters of spheres that can be located in the systems’ voids (further details in Section S1.3.3). The PDF of each amorphous phase (*G*(*r*)) was calculated using the RMCProfile software package^70^ and is reported in the *D*(*r*) form, obtained as *D*(*r*) = 4π*r*ρ*·G*(*r*). Further details on the procedure used for the PDF calculation and the application of principal components analysis on it are reported in Sections S1.3.4 and S1.3.5. The PDFs calculated from our computational models were compared to experimental PDFs taken from ref (63).

We exploited the versatility of Polymatic to build nine different amorphous phases with different degrees of maintained local order (by changing the initial building blocks used) and defects (by changing the degree of polymerization). For each phase, the results are averaged over five independent models formed from different initial random packings, meaning that a total of 45 models are studied in this work, of which 15 were published previously.^33^ All structural models are available at https://github.com/Ibechis/FeBTC_models.

## Results and Discussion

### Phase Space of the Amorphous Models of Fe-BTC

Using our approach, we explore amorphous phases with Fe-BTC composition at three levels of maintained local order and three levels of defects. The level of local order, which we simply refer to with the terms “order” or “disorder” here, is controlled by the initial building blocks used for structure generation, with the trimers (Figure 2i) defining the local order of the system in the short range and the tetrahedra (Figure 2ii) defining the local order of the system in the medium range. In the most disordered models, only trimers and BTC linkers are used as initial building blocks (SRO phases). Phases at higher degrees of order include half of the trimers as “free”, the other half organized in the tetrahedra arrangement, and BTC linkers (MIX phases). Phases at the highest level of order are built from a mixture of tetrahedra and BTC linkers (MRO phases). If tetrahedra are not present at the beginning, none are likely to form during the following polymerization. The defect content is controlled by the degree of polymerization of the structure, with each missed bond corresponding to the introduction of a defect. We define a defect as the combination of an ML defect (two adjacent iron atoms on a trimer saturated with H_2_O and OH groups) and an MN defect (an uncoordinated COOH group on a BTC linker), arising from the missed bond that would have connected the two reactive sites. To obtain the lowest level of defects, we let the polymerization run until no further reactive pairs within the established cutoff could be found, reaching a percentage of polymerization higher than 90% of the total amount of bonds that could be formed if every pair reacted and generating final models with less than 10% of possible defects. To create structures that contain more defects, we stopped the polymerization at 80 and 70%, creating two additional sets of models with 20 and 30% of defects, respectively, for each of the degrees of order (SRO, MIX, and MRO). In our models, 100% of polymerization corresponds to the formation of 2400 bonds; therefore, structures at 20 and 30% of defects have around 480 and 720 missing bonds, respectively (Table S8).

**Figure 3:**
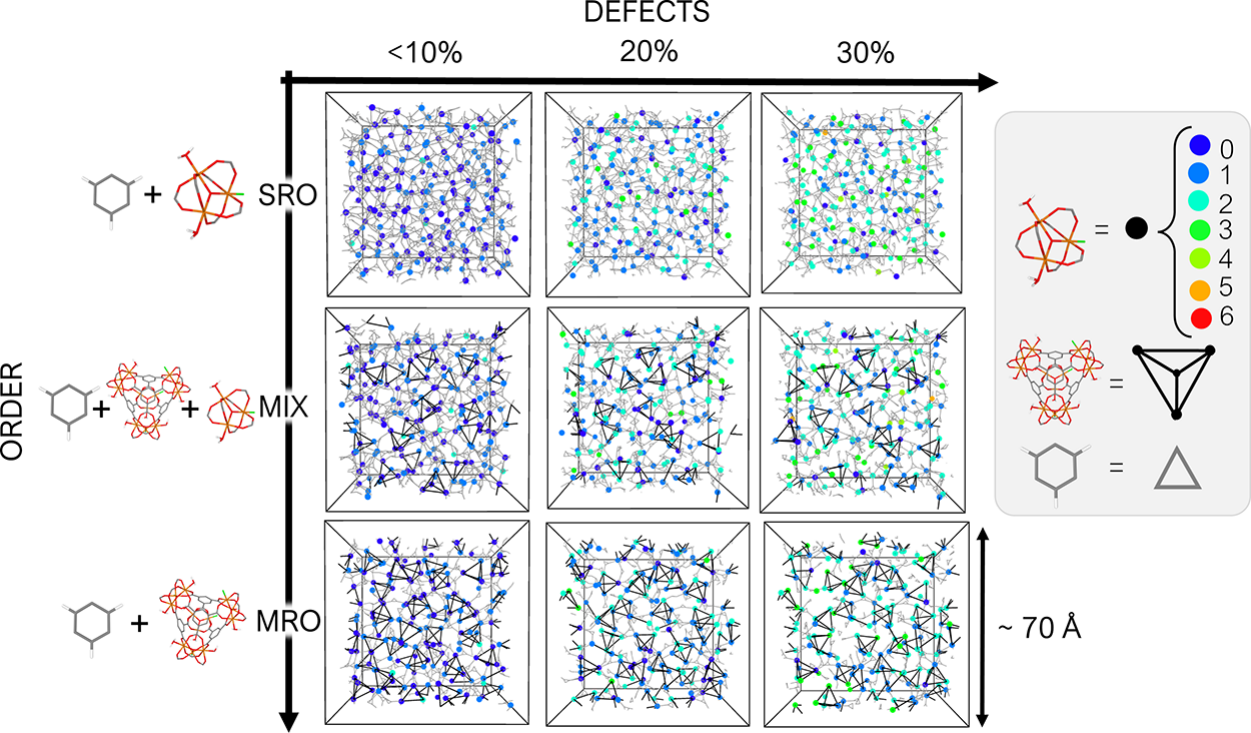
Phases studied in this work. On the *y*-axis, the level of order is controlled by the building blocks used in the random packing (SRO = trimers + BTC linkers; MIX = trimers + tetrahedra + BTC linkers; MRO = tetrahedra + BTC linkers), while on the *x*-axis, the level of defects can be controlled by the degree of polymerization. A slice of one model for each of the nine phases is shown in a schematic representation (legend on the right), in which trimers are reported as circles, color-coded by the number of MLs as a measure of the defect content in the structure, tetrahedra as black tetrahedral shapes, and linkers as gray triangles. All studied models are periodic.

The nine different generated amorphous phases are schematically represented in a two-dimensional space in Figure 3, where the degree of defects is on the *x*-axis and the degree of order is on the *y*-axis. SRO, MIX, and MRO structures with less than 10% defects have already been published in our previous work together with their calculated PDF, PSD, and N_2_ surface area.^33^

### General Characterization of the Amorphous Models

We now present the results of the amorphous structure generation workflow, in terms of degree of polymerization reached, density, and defect distribution. We remind the reader that no experimental data were used during the structure generation, making this a predictive approach. When allowed to reach completion, the polymerization reaches 91–92% for all the disorder levels (Table 1). This is lower than previous studies on *a*ZIF-4^30^ where the polymerization would reach ∼98%, which can be explained by the hindered movement of the bigger building blocks of Fe-BTC compared to ZIF-4 (Zn atoms and imidazolate linkers) that decreases the likelihood that two reactive sites will be close to each other during the polymerization phase and bond formation. The final models, after saturation and annealing, have around 20,000 atoms and have an average cell parameter of ∼70 Å (cell volume of ∼343,000 Å^3^) (Table S8).

**Table 1:** Results of the Amorphous Structure Construction Workflow^a^

order	SRO			MIX			MRO		
defects	<10%	20%	30%	<10%	20%	30%	<10%	20%	30%
% polymerization	92.1 (1.0)	80.0 (0.1)	70.0 (0.1)	92.2 (0.5)	80	70	91.5 (0.3)	80	70
final bulk density (g cm^–3^)	1.49 (0.02)	1.43 (0.02)	1.40 (0.02)	1.41 (0.02)	1.34 (0.00)	1.31 (0.01)	1.23 (0.02)	1.15 (0.01)	1.16 (0.02)
final skeletal density (g cm^–3^)	1.57 (0.01)	1.48 (0.01)	1.43 (0.01)	1.51 (0.01)	1.43 (0.00)	1.39 (0.00)	1.45 (0.01)	1.36 (0.00)	1.33 (0.01)
number density (Å^–3^)	0.0670 (0.0005)	0.0669 (0.0011)	0.0678 (0.0011)	0.0631 (0.0009)	0.0628 (0.0002)	0.0637 (0.0003)	0.0551 (0.0007)	0.0541 (0.0006)	0.0561 (0.0010)

a Reported values for each phase are the average over the five independent models, with standard deviations in parentheses.

To analyze how changing the defect concentration and the extent of disorder in the structure affect the density of the system, both the skeletal and bulk densities were calculated (Table 1). The skeletal density was measured by subtracting the accessible volume to a probe with a diameter of 2.6 Å (equivalent to the kinetic diameter of helium)^71^ from the total volume, thus providing density values more comparable to experimental ones obtained by helium pycnometry. The standard deviation of the final bulk and skeletal densities among the five independent models for each phase is low (lower than 0.02% of the average value), suggesting a good agreement between the five models and an overall good statistical sampling of the systems. The density of the systems varies with both the degree of disorder and defects, decreasing with the increasing number of defects and increasing with the introduction of disorder. This trend is also evident from both the bulk and the skeletal densities for the SRO and MIX series, while for the MRO series, the effect is more marked when looking at the skeletal density. The notable difference between the bulk and the skeletal densities obtained for the MRO series compared to the SRO and MIX configurations suggests that MRO structures are more open and have more volume accessible to a helium-sized probe. This is perhaps intuitive, given that the packing of tetrahedra will be less efficient than the connectivity of simple trimers, therefore leading to accessible space within the structure.

In defect studies on crystalline MOFs, it has been shown that defects are randomly distributed at low concentration, but correlated and clustered in defective regions at high concentration.^9^ It may be interesting in the future to study how the phenomenon of correlated defects translates into an amorphous system at high concentration. This could be achieved by introducing an additional energetic-based rule to the geometric one used for bond formation. Alternatively, one could selectively cap reactive sites or assign them opposite charges of different values during polymerization. In this way, the bond formation can be biased toward the desired outcomes. However, with the current approach, we did not control where the missing bonds occur in the network during polymerization and, therefore, the location of the defects in the final model is arbitrarily distributed.

**Figure 4:**
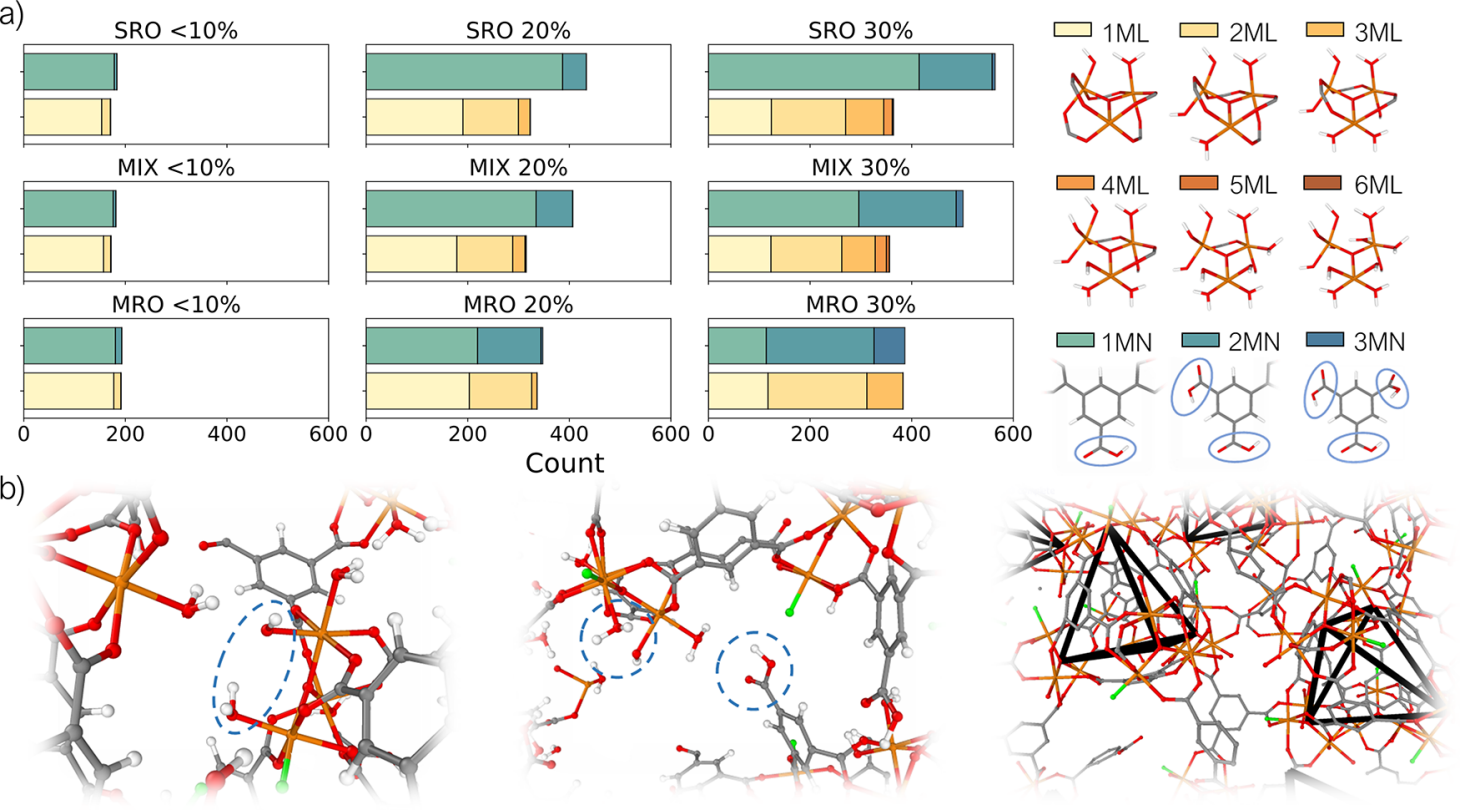
(a) Distribution (left) and definition (right) of the defect types (ML = missing linker and MN = missing node) in the nine different phases studied. The distribution reports the average number of defect types over the five models for each phase. The relative position of the ML defects in the clusters used for defect definition is arbitrary. Terminal water molecules and F atoms have been omitted for clarity. Carbon is shown in gray, oxygen in red, iron in orange, fluorine in green, and hydrogen in white. (b) Example of defective structures (left and center, circled in blue) and local order maintained by tetrahedra (right, hydrogens removed for clarity, tetrahedra highlighted in black) in the *a*MOF models. The snapshots only show a slice of the models for clarity.

A “free” trimer may lack up to six linkers, while a trimer included in a tetrahedron can miss up to three linkers, in what we define as ML-type defects (Figure 4a). A free BTC linker can miss up to three trimers in what we define as MN-type defects (Figure 4a), while BTC linkers that were included in a tetrahedron from the initial random packing step are not able to change their connectivity and host defects in the following polymerization step. Figure 4a reports the distribution of these types of defects in the nine studied phases. As expected, the overall number of defective nodes and linkers increases when considering the series from 10 to 20 to 30% of defects. Since tetrahedra substructures can only host up to three ML defects on their trimers, the presence of highly defective structures (i.e., >3 ML defects on the same node) can only occur in the SRO and MIX phases. Even in these systems, completely defective nodes (6ML defects) are never observed, and 5ML and 4ML defects are rare. Increasing the order in the structure from SRO to MIX to MRO increases the number of 2MN or 3MN defects (the latter corresponding to completely unreacted linkers). In the MIX and MRO systems, a high number of linkers are involved in tetrahedra, and additional defects can only be placed in the remaining “free” linkers. When keeping the level of order constant and increasing the percentage of defects (<10 to 20 to 30%), progressively more defective building blocks can be found in the structures (i.e., higher numbers of 2ML, 3ML, and 4ML nodes and 2MN and 3MN linkers) for all the phases.

Examples of defective structures inside the models are reported in Figure 4b. The presence of these undercoordinated species in Fe-BTC models is not unrealistic, although we note that our approach does not include any energy-based assessment for defect formation that guides which of these types of multiple defects forms. Experimental infrared spectroscopy studies on both Fe-BTC and *a_m_*MIL-100 have confirmed the presence of structural OH groups belonging to BTC linkers binding in a monodentate fashion or completely unreacted.^33,50,54,55,63^ On the metal, these ML defects are likely to become coordinated by atmospheric water, whose presence has been confirmed by thermogravimetric analysis for both Fe-BTC and *a_m_*MIL-100, which exhibit between 2 and 4 wt % loss associated with structural water molecules.^33,63^

### Geometric and Energetic Analysis of the Amorphous Models

**Figure 5:**
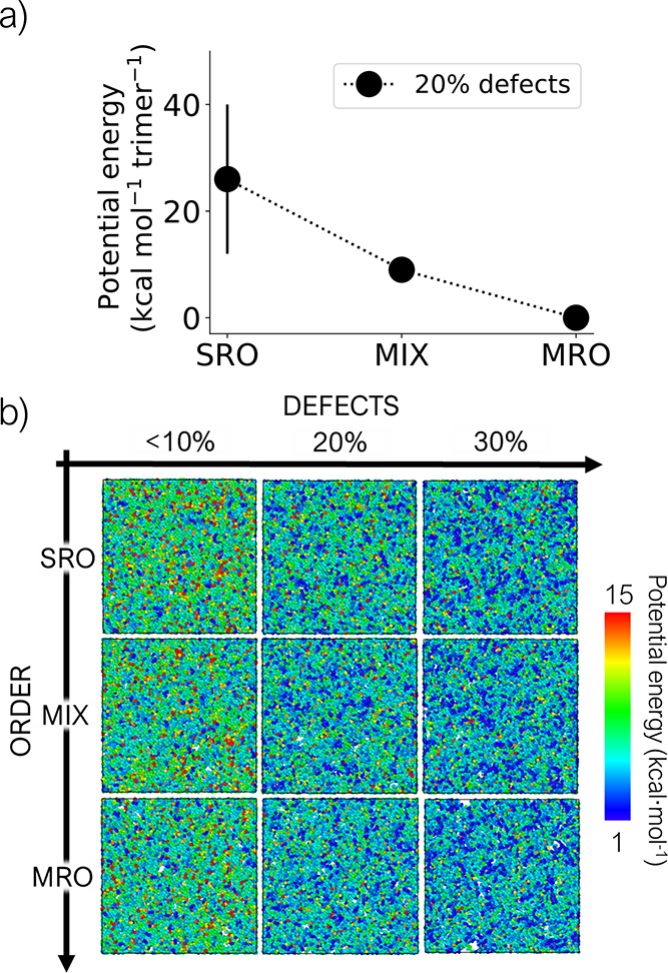
(a) Potential energy values per trimer (average over the five models with standard deviations as error bars) for models at 20% of defects and different levels of order. Other levels of defects are reported in Figure S6. (b) Visualization of a representative model for the nine studied phases, with individual atoms colored according to their potential energy. Hydrogen atoms and groups in terminal positions of the iron atoms (H_2_O and F) have been removed to highlight changes in nodes and linkers.

We now look at how the presence of defects and disorder manifests in the microstructure and potential energy of the models (further details in Sections S1.3.1–1.3.2). We are aware of the general nature of UFF as a force field and the absence of a well-defined reference value for UFF geometry. Therefore, we focus on a qualitative analysis of the energetics of the models, in particular, looking at how the ranges of relative energies changes for different models. The effect of increased order (i.e., inclusion of an increased proportion of tetrahedra in the structure) can be observed from structural features such as radial distribution functions (increased peak intensity around 10 Å, the MRO region, Figure S7) or the distance and orientation between pairs of BTC linkers (with distributions centered around specific values for tetrahedra-containing phases, Figure S8). From an energetic point of view, increasing the degree of local order in the structure corresponds to lower average potential energy per trimer (Figure S6). Atoms in the MRO phases always have lower energy, on average, than in the MIX and SRO phases when compared at the same level of defects (at 20% defects, SRO 20% > MIX 20% > MRO 20%, as reported in Figure 5a).

To compare the energy of systems at different levels of defects, we analyzed the force field potential energy of each atom in the systems. By color-coding the atoms according to their potential energy, as done in Figure 5b, it is visually clear that atoms in more defective structures have lower energy. We believe that this is related to the lower degree of crosslinking between building blocks in more defective models, which results in less strained models. In particular, by visual inspection of the models, we find that the most strained substructures at lower levels of defects are iron atoms on trimers (Figure S9), which lose their strain when one or more linkers are missing and are substituted by “free” (not interconnected) OH/H_2_O groups in more defective models. This relaxation is reflected in several other structural features, including angles, dihedrals, RDFs, and distance and orientation between pairs of BTC linkers (Figures S7–S11). All these metrics show narrower peaks in their distributions when comparing models with the increasing level of defects, but constant level of order, suggesting that their building blocks are adopting more uniform (and less distorted) arrangements, as a consequence of the reduced strain in the system.

### Porosity of the Amorphous Models

To characterize the porosity of the amorphous systems, we calculated several geometric measures of porosity with Zeo++.^69^ The analysis is purely structural, as the systems are kept rigid during the calculation and no real adsorbate is introduced in the model. Sometimes, computationally obtained values for porosity in MOFs from their rigid crystal structures underestimate the experimental results because the flexibility of the framework and the consequent swelling caused by the guest adsorption are not considered in the calculation. As an example, the N_2_ accessible surface area of MIL-100(Fe) calculated by Zeo++, for which the crystalline structure is known, is 1687 m^2^ g^–1^, while experiments report a surface area of 2240 m^2^ g^–1^ by N_2_ adsorption.^33^ Assuming that the degree of swelling will be similar for disordered materials with the same building block chemistry, we look at trends in porosity across our model series. In the future, it will be interesting to investigate the nature and the extent of framework flexibility and swelling behavior in disordered systems and the role of local or long-range disorder.

**Figure 6:**
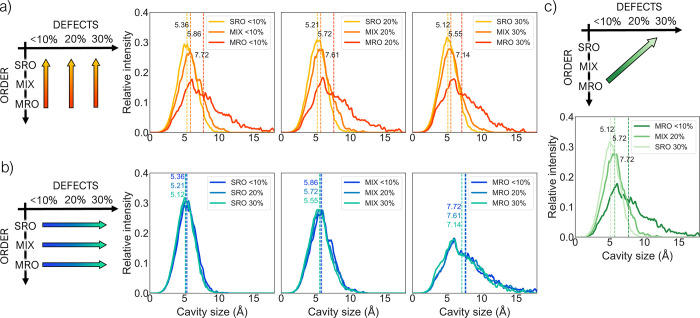
PSDs for models at (a) the same level of defects but different levels of disorder and (b) the same level of disorder but different levels of defects. (c) PSD of models moving along a diagonal in the phase space: the level of disorder and defects in the systems are changed simultaneously. The arrows across the schematic phase space help understand which three phases are reported together in the adjacent plots. The reported PSD for each system is averaged over the five independent models. Average cavity size values for each distribution are reported as dotted lines.

PSDs for different phases are plotted together to highlight trends when disorder or defects are increased independently (Figure 6a and Figure 6b, respectively) or simultaneously (Figure 6c). As would be expected, disorder has a much greater influence compared to defects on the PSD of the system. All systems have a peak for the cavity size centered around 5.0–5.5 Å, but MIX and SRO structures show pores up to 10.0 and 11.0 Å in diameter, respectively, while MRO structures have pores with diameter up to 17.0 Å. Defects, on the other hand, have a very small impact on the PSD, leading to only a slight decrease in the average cavity size for more defective structures. When defects and disorder are introduced in the system simultaneously, the overall effect is a shift to lower pore size values in the PSD, mainly controlled by the increase in disorder.

**Figure 7:**
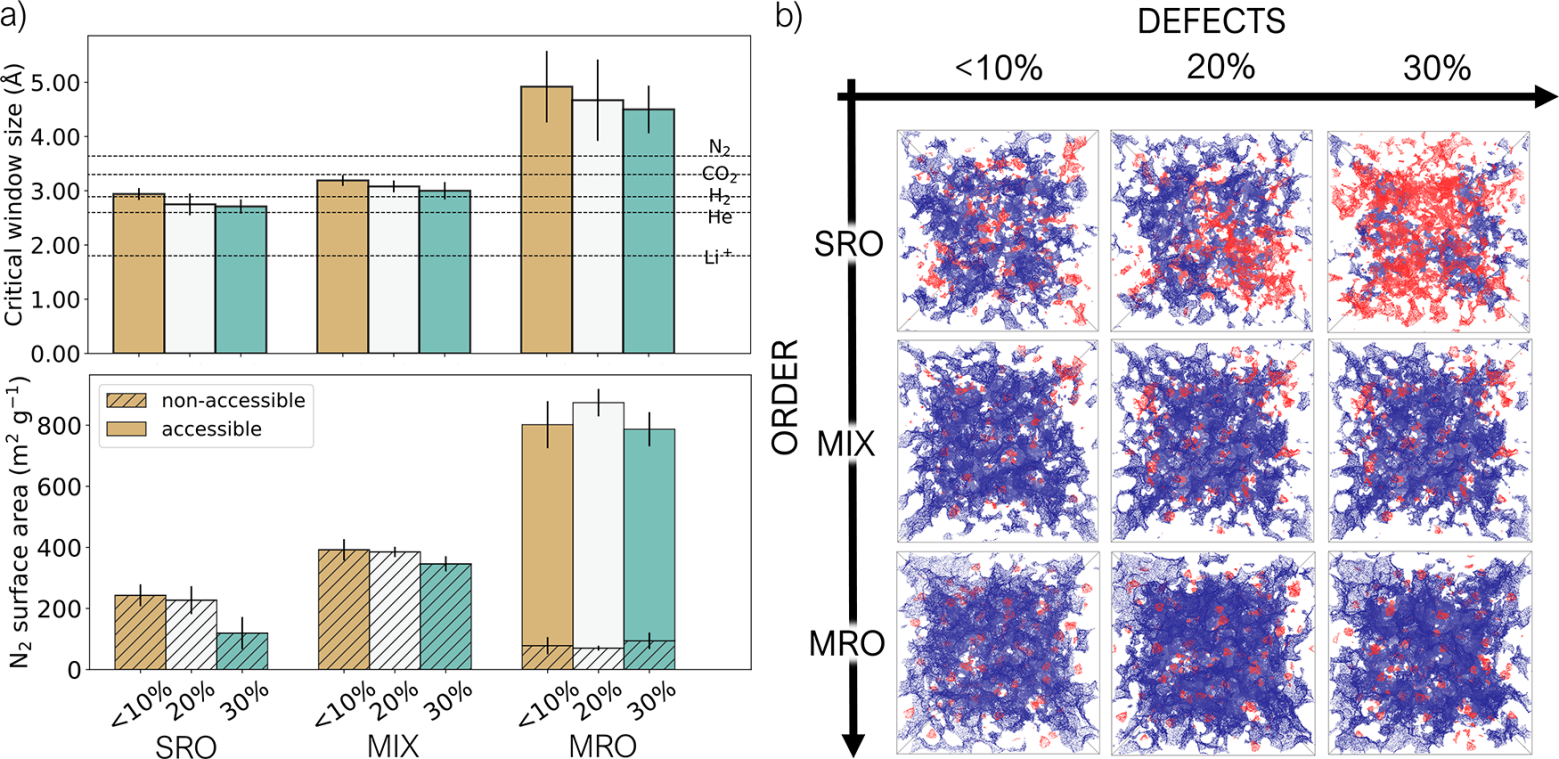
(a) Top: average critical window size. Bottom: average accessible (plain) and non-accessible (hatched) surface area calculated with a probe of 3.64 Å in diameter (kinetic diameter of a N_2_ molecule) for the nine studied phases. Standard deviations over the five independent models are reported as error bars. (b) Accessible (blue) and non-accessible (red) surface area for a probe size of 2.60 Å in diameter. Only one representative model is reported for each phase.

Other useful measures of the porosity of a system are the diameter of the largest sphere that can be included in the model (*D*_i_), the diameter of the largest sphere that can percolate through the model (*D*_f_ or critical window size), and the diameter of the largest sphere accessible along the path of *D*_f_ (*D*_if_) (Figures 7a, S12a, and Table S9). As seen for the PSD, we found that the effect of disorder is much larger than the effect of defects: increasing the level of disorder reduces all the studied measures of porosity. The effect of defects is less clear, and variations between phases at the same level of order but different levels of defects easily fall into the standard deviation of the individual phase.

The critical window size (diameter of the biggest sphere that can percolate through the model) allows us to speculate on what guests the material is porous to. The plot in Figure 7a reports the kinetic diameters of some guests compared to the critical window size of the nine amorphous phases. All the systems would be accessible to a probe of the size of He or H_2_ (kinetic diameter of 2.60 and 2.89 Å, respectively),^71^ MIX models are slightly accessible to CO_2_ too, while MRO models are accessible to both CO_2_ and N_2_ (kinetic diameter of 3.30 and 3.64 Å, respectively).^71^ All the models still present a portion of non-accessible surface area occupiable by a probe with the dimension of a N_2_ molecule, as can be seen in the bottom plot of Figure 7a, with the trends in the total surface area (accessible and non-accessible together) reflecting the expected increase with the increased order. Only the MRO models are accessible to N_2_ by this analysis, with average accessible surface area values of 724(77), 804(45), and 693(56) m^2^ g^–1^ for the 10, 20, and 30% defective structures, respectively. A portion of the non-accessible surface area for the tested probes is expected to become accessible when framework mobility and swelling caused by adsorption are considered, especially for those probes whose diameter is close to the obtained *D*_f_ values for the models. In these cases, only a small amount of motion would be required to increase the window size and make the structure accessible (e.g., CO_2_ for the MIX phases).

The slight decrease of critical window size values with increasing defects suggests a reduction in accessibility of the pores in the structure when defects are introduced, which can be explained by the presence of capping groups partially hindering the aperture of some of the channels in the structure. Reduced accessibility to pores is confirmed by the evolution of the accessible surface area in the systems as a function of probe size (Figure S12b). This metric shows that for probe diameters <2.00 Å, the increase in the number of defects leads to higher values of accessible surface area, meaning that when the probe is small enough to access all the pores even in defective structures (that could be, for example, Li^+^ with a kinetic diameter of 1.80 Å),^30^ defects actually increase the available surface area. We suggest that more defective structures have narrower channels and, therefore, less accessible pores, but still show interesting porosity for small probes like ions. For probes bigger than 2.00 Å, only small differences are observed in the values of accessible surface areas as a function of defects (with the exception of SRO systems, where increasing defects decreases the accessible surface area) and is reported visually in Figure 7b, which shows the accessible (in blue) and non-accessible (in red) surface area for a probe of 2.60 Å (kinetic diameter of He) in one model for each phase. Looking at the systems that incorporate tetrahedra, it is clear that the void inside the tetrahedra is included in the non-accessible surface area portion (red spots in Figure 7b). Thus, the porosity of the systems that include tetrahedra is not coming from the intrinsic porosity of these cage-like structures, but from the external voids left by their disordered packing.

### Analysis of the Pair Distribution Functions of the Amorphous Models

**Figure 8:**
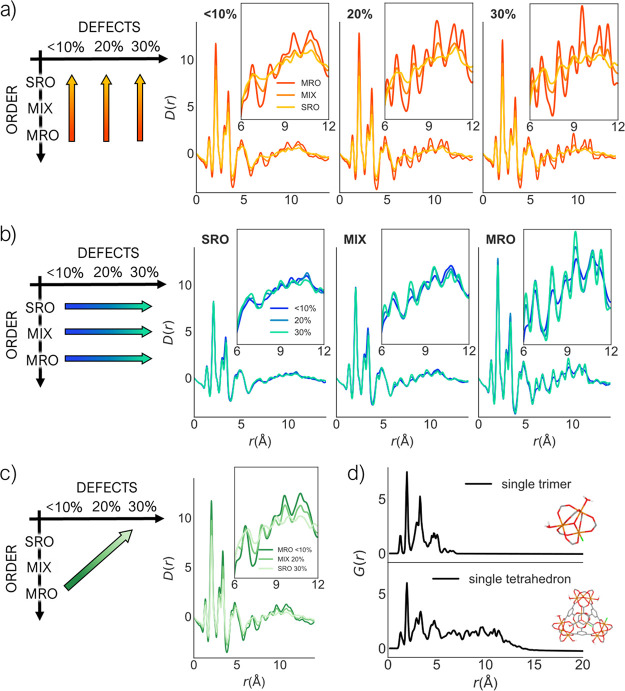
Calculated PDFs for one representative model of each phase at (a) same level of defects but different levels of order and (b) same level of order but different levels of defects. (c) Calculated PDFs of models moving diagonally in the phase space, changing the level of disorder and defects simultaneously. Insets show a zoom-in on the 6–12 Å range. The arrows across the schematic phase space help understanding which three phases are reported together in the adjacent plots. (d) PDF of a single trimer (top) and a single tetrahedra (bottom).

Total scattering techniques, and in particular PDF, have been increasingly used to investigate the structure of *a*MOFs. Experimentally collected PDFs contain information on the local structure within the material, and as such, comparing calculated PDFs of structural models to experimental data is a useful tool for obtaining atomistic-level structural understanding. We calculated the PDFs of our models (Figure 8) to understand the influence of different factors (the degree of defects and disorder) on their main features.

We plot together the PDFs highlighting the trends when either disorder or defects are varied independently (Figure 8a and Figure 8b, respectively) or simultaneously (Figure 8c). We report the PDF for one model for each phase, after assessing that there are negligible differences between PDFs of the five replicas (Figure S13). All of the PDFs exhibit similarity due to the similar local structures and are broadly comparable to our previous in-depth study on the experimental PDF for Fe-BTC.^33^ Noting that individual partial PDFs are weighted more strongly in the total PDF if they contain heavier elements and/or in a higher concentration, the first three major peaks in the PDF are largely dominated by the Fe–O, Fe–Fe, Fe–C, and C–O pairwise interactions (Figure S14). More generally, observable peaks between 0 and 7 Å largely arise from the oxo-centered trimer motif, while those between 7 and 12 Å are predominantly from the assembly of the trimers into the tetrahedral units (Figure 8d). Beyond 12 Å, identifiable peaks in the PDF represent spatial ordering of the tetrahedral units. The calculated PDFs of all nine models are largely featureless beyond 12 Å due to their amorphous nature. The polymerization step forms bonds between the building blocks; however, it does not affect the structure of the building blocks themselves. Hence, during the simulation, the structure of these building blocks remains essentially the same and hence minimal variation is observed in the low-*r* region of the PDF.

Figure 8a highlights the effect of changing the disorder level when the defect level is kept constant. The most significant variation in these series occurs in the 7–12 Å region and is attributed to the varying proportion of tetrahedral units, which give rise to correlations in this region. The intensity of the peaks in this region increases SRO < MIX < MRO. Furthermore, the addition of tetrahedral units induces appreciable variations in the distribution of atom density within a single model, with “empty” regions interspersed with denser clusters of atoms. These fluctuations in atom density give rise to additional scattering at low-*Q* for tetrahedra-containing systems (Figure S15). This additional scattering can manifest as a low frequency oscillation of low intensity that modulates the fluctuating baseline of the PDF and can contribute to the overall increase/decrease of PDF peak intensities.^72,73^ Incorporation of tetrahedral units also reduces the overall atomic number density of the system, which plays a role in the scaling of the PDF.^74^

Figure 8b highlights the effect of changing the defect level when the disorder level is kept constant. Here, the variation is significantly less than that shown in Figure 8a, suggesting that defects have less impact on the structure than the degree of disorder. This is understandable as the low-*r* region of the PDF is dominated by the structure of the building units, which have well-defined geometries that essentially stay the same throughout the simulation. Interactions between the building blocks primarily occur beyond this low-*r* region: trimer–trimer distances are approximately 10 Å and thecentroid distance between benzene rings is around 6.5 Å. Differences in the low-*r* region do exist due to the presence of defects; however, they are only subtle and largely masked by the dominant contribution from the local structure of the building units. These minor variations arise from a combination of different subtle factors. First, the increased concentration of oxygen atoms in the models, which results from the increasing number of capping OH/H_2_O ligands required to saturate systems with the increasing defect content, leads to an accentuation of all oxygen-containing partial PDFs (and in particular the Fe–O partial PDF, which dominates the total PDF). Second, the previously mentioned fluctuations in atom density and changes in overall atomic number density will contribute minor variations in the PDF. Finally, the relaxation of less strained tetrahedra in defective structures may also play a role in the intensity of the peaks in the 7–12 Å region for MIX and MRO phases.

In Figure 8c, we consider the structural effect of simultaneously varying the level of disorder and defects, specifically reducing the proportion of tetrahedra while increasing the number of defects (i.e., MRO 10% > MIX 20% > SRO 30%). In changing both the level of disorder and defects, all of the aforementioned factors (low-*Q* scattering, oxygen concentration, density, and so on) will simultaneously affect the PDF. Visual inspection of the series in Figure 8c reveals that the majority of the variation occurs in the 7–12 Å region, originating from the change in proportion of tetrahedra. As already seen for porosity and for PDF trends at constant defect levels, this result suggests that it is the level of disorder, and not defects, that has the strongest influence over the structure of the models.

To better visualize the way in which defects and disorder affect the PDFs, we adopted our recently reported multivariate analytical approach^72^ to probe our series of PDFs (details in Section S1.3.5). Applying principal component analysis, a dimensionality reduction technique, on a series of PDFs, we can extract two statistically significant principal components from the series. The first resembles the typical form of a PDF and describes the common atom–atom correlations within the series, while the second, the distortion component, describes the way in which the PDFs deviate from the statistical average of the data set (Figure S16a). From the distortion component, we can deduce the nature and extent of the structural distortion that is occurring across a PDF series (Figure S16b). The distortion PDFs obtained from the series at the constant defect level (i.e., increasing proportion of tetrahedra) have a characteristically similar increasing envelope of the peak intensities. In all three distortions, the intensity of the peaks increases up to 12 Å, after which the distortion is largely featureless. This is a direct visualization of the additional correlations that appear, particularly in the 7–12 Å region, when the proportion of tetrahedra in the system increases. The highest intensity peak around 10 Å correlates with the emergence of well-defined trimer–trimer distances, as observed in Figure S7 and the subsequent Fe–Fe correlations that arise from this. On the other hand, the distortion PDFs obtained from the series at the constant level of order (i.e., increasing proportion of defects) show a far more uniform envelope of the peaks in the 0–12 Å region, indicating that a similar extent of structural distortion occurs throughout the local structure. Two exceptions to this uniform shape are the high intensity peaks at 1.86 and 3.24 Å, which capture changes in the peak positions.^72^ The former captures changes in the Fe coordination sphere from the Fe–O bonds, while the latter captures changes in the peak dominated by Fe–Fe and Fe–Fe correlations. The distortion PDF obtained for the diagonal trend corresponding to simultaneous change in defect and local order is intermediate to those just described.

### Comparison with Experiments

Even as simplified representations of real Fe-BTC phases, our models can be used to help interpret and unify the range of experimental data reported for Fe-BTC materials and provide atomic scale insights that are not possible through experimental characterization alone. Crucially, we establish that it is the level of order within the local structure, which has the greatest effect on the structural properties of Fe-BTC materials and not the degree of defects. We find that a key structure–property relationship exists between the proportion of tetrahedra within the system and its accessible surface area. This relationship can help to rationalize the diverse range of experimental BET surface area values obtained for different Fe-BTC systems within the literature, with the various synthetic procedures resulting in different proportions of trimers and tetrahedra. Through comparing the calculated structural properties to those observed experimentally, we can begin to assign different Fe-BTC materials to the different models, as shown in our previous work,^33^ where the MIX (<10%) and MRO (<10%) phases were assigned to a sol–gel-based Fe-BTC material and Basolite F300, respectively. Now, with the additional nuance of defects within our models and given the propensity for Fe-BTC systems to contain defect sites, we are able to develop an even deeper understanding of the structural chemistry that underpins this functional group of materials.

**Figure 9:**
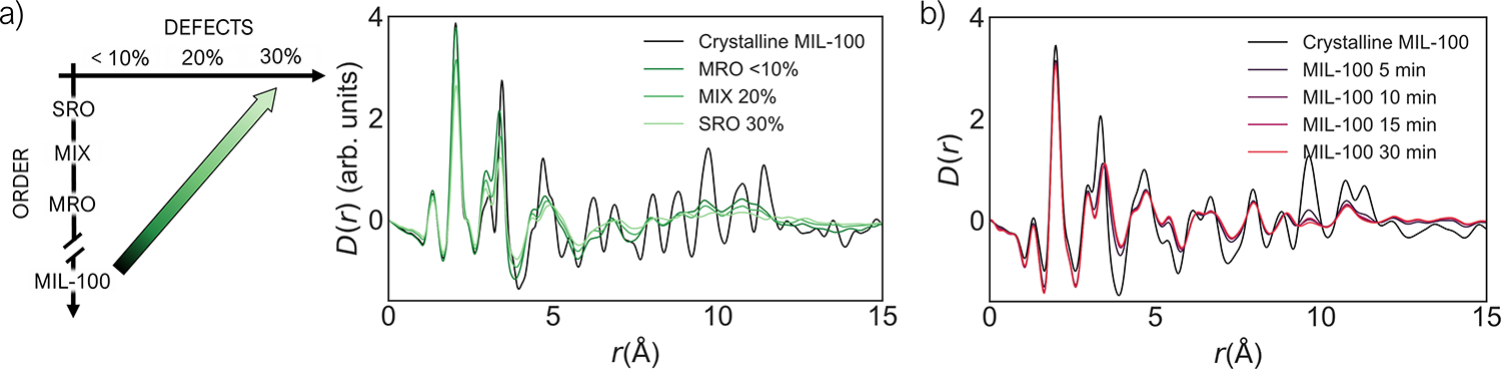
Comparison between (a) calculated PDFs of crystalline MIL-100 and amorphous models with simultaneous increase of defects and disorder (corresponding to moving diagonally in the phase space defined in this work, as shown by the green arrow on the left) and (b) experimental PDF of MIL-100 at different ball-milling times from ref (63). Computed PDFs have been rescaled to be comparable with experimental PDFs.

Not only do our models provide a basis for the complex structures of Fe-BTC materials, but they aid our understanding of the crystalline MIL-100 material and its structural collapse upon ball-milling. PDF analysis performed by Sapnik et al. following the structural collapse of MIL-100 over the course of 30 min to form *a*_m_MIL-100 suggested that ball-milling breaks metal–linker bonds, causing a reduction in the number of tetrahedral units.^63^ Hence, ball-milling leads to a simultaneous change in the level of defects and disorder. With this in mind and with the additional control over the proportion of defect sites in the models generated here, we can tentatively ascribe the structural transition that occurs during ball-milling of MIL-100 as analogous to the diagonal trend studied here, with the crystalline structure of MIL-100 as the starting point. Figure 9 reports a comparison of the calculated and experimental PDF series for these two structural evolutions. From a visual comparison, we can see that there is reasonable agreement between the trends observed in both series. We note that there would be a reasonable agreement also with vertical trends, as the main changes caused by ball-milling that are visible on the PDF involve changes in disorder, but the diagonal trend is the only one that accounts also for the increase in defects highlighted experimentally by TGA and IR data.^63^ This computational insight represents the first steps toward understanding the mechanism of structural collapse in MIL-100 from an atomic perspective. From an applied perspective, this enables us to investigate the properties of these partially collapsed materials and also to help better address the industrial challenges regarding collapse of MOFs within practical applications.^13^ Together, this analysis exemplifies how our computational approach can offer an insight into the mechanism of framework collapse and *a*MOF formation.

### Limitations of the Presented Approach

Disorder/defect modeling in MOFs is still in its infancy compared to crystallographic structure solution and this is a first step toward bridging this gap. The goal of this polymerization-based approach is to provide a relatively fast and reliable method to explore and test new phases of *a*MOFs, from which useful design rules can be drawn. This approach does not aim at providing an accurate description of the phase formation mechanism, which is sometimes very far away from the polymerization-like approach adopted (e.g., melt-quenching). Mechanistic insights on MOF formation are currently only accessible through computationally expensive ab initio methods. Here, insights on ball-milling effects were obtained by combining computational and experimental results and only to confirm a mechanism previously suggested. Another limitation of this approach in the current implementation is the lack of an energetic criterion to satisfy during bond formation, which sometimes results in the formation of strained structures (intermediate MD cycles during polymerization aim to reduce this strain). Force fields are, overall, still the biggest limitation in describing these disordered systems. The complexity and size of the system requires a classical approach, but the description of the coordination bond at a classical level is challenging, even in the study of crystalline MOFs. In the case of *a*MOFs, an additional challenge is posed by the fact that MOF force fields are derived using crystalline structures as reference geometries and not tested to represent the distortions present in amorphous materials. In addition, we remind the reader that all calculations have been performed without solvent, which for some systems, like *a*MOF gels directly synthesized from solution, could play an important role in the structure formation.

## Conclusions

We have exploited the versatility of a polymerization-based algorithm to model nine amorphous phases with the composition of Fe-BTC, with varying degrees of defects and disorder. We find that disorder has a much stronger influence on the porosity of the systems, with a higher degree of accessible porosity and bigger pores observed in structures that maintain MRO features. Contrary to what has been observed for defect engineering in crystalline MOFs,^10^ defects in amorphous Fe-BTC appear to have a negligible effect on structural porosity, although we expect them to affect catalytic activity or adsorption properties due to the increased number of active sites.^75^ More generally, this study suggests that extending the degree of local order, or the use of rigid and larger building blocks (such as tetrahedra) that pack inefficiently, can be used as a strategy to introduce permanent porosity in *a*MOFs. Similar approaches have been adopted to increase porosity in purely organic amorphous materials, such as using molecules with internal cavities (similar to the tetrahedra in this study) in porous molecular materials^76−78^ or rigid monomers that impede efficient packing in polymeric materials.^79,80^ Because of the high coordination number of metal ions and the complex geometries subsequentially available, MOFs are good candidates for the control of the degree of local order via hierarchical substructures. Therefore, we suggest that other crystalline MOFs that show a hierarchical order in their net topology, such as MOF-500 (which includes two different types of tetrahedral subunits) or some DUT MOFs (which includes octahedral and cuboctahedra subunits), could be good candidates for obtaining *a*MOFs with controlled permanent porosity.^81^

This modeling approach gives us the unique opportunity to disentangle the individual effects of defects and disorder. Experimentally, we are not afforded with the same control. This work highlights the necessity for new experimental approaches to introduce defects or disorder into MOFs independently of each other. While defects and disorder are intimately connected, there has been significant research in the area of “defect engineering” in crystalline MOFs.^10^ Here, our work suggests that “disorder engineering” could have an equally, if not more, important role to play in the field of non-crystalline materials. Experimentally, strategies toward disorder engineering could use preassembled building units (like metal–organic polyhedra) in *a*MOF syntheses. Computationally, the current approach could be used to explore disorder at higher length scales by using larger preconfigurations of building blocks.

The presented computational approach is versatile not only toward modeling phases with varying structural features at fixed chemical composition but also in terms of chemistries that can be covered. We can envisage this approach being used in a systematic way to explore a high number of *a*MOF structures, where the degree of freedom can be the linkers or the nodes (also in a mixed fashion) and not just the degree of disorder and defects. The independence of this approach from an initial crystalline structure or any experimentally derived data for structure refinement makes it an excellent candidate for exploration of hypothetical structures. Access to realistic models for amorphous materials enables their collection in structural databases and opens up the possibility of exploiting the collected knowledge toward design of new materials or screening of the existing ones for selected applications, similar to approaches developed for crystalline materials.

## Supporting Information Available

Additional information on force field validation, amorphous structure construction procedure, and additional structural and porosity analysis (PDF)

## References

[ref1] Yaghi O. M.; O’Keeffe M.; Ockwig N. W.; Chae H. K.; Eddaoudi M.; Kim J. Reticular Synthesis and the Design of New Materials. Nature 2003, 423, 705–714. 10.1038/nature01650. 12802325

[ref2] Furukawa H.; Cordova K. E.; O’Keeffe M.; Yaghi O. M. The Chemistry and Applications of Metal-Organic Frameworks. Science 2013, 341, 974 10.1126/science.1230444. 23990564

[ref3] Shen Y.; Pan T.; Wang L.; Ren Z.; Zhang W.; Huo F. Programmable Logic in Metal–Organic Frameworks for Catalysis. Adv. Mater. 2021, 33, 2007442 10.1002/adma.202007442. 34050572

[ref4] Bennett T. D.; Coudert F.-X.; James S. L.; Cooper A. I. The Changing State of Porous Materials. Nat. Mater. 2021, 20, 1179–1187. 10.1038/s41563-021-00957-w. 33859380

[ref5] Bennett T. D.; Cheetham A. K.; Fuchs A. H.; Coudert F.-X. Interplay between Defects, Disorder and Flexibility in Metal-Organic Frameworks. Nat. Chem. 2017, 9, 11–16. 10.1038/nchem.2691. 27995920

[ref6] Cheetham A. K.; Bennett T. D.; Coudert F.-X.; Goodwin A. L. Defects and Disorder in Metal Organic Frameworks. Dalton Trans. 2016, 45, 4113–4126. 10.1039/C5DT04392A. 26836459

[ref7] Xiang W.; Zhang Y.; Chen Y.; Liu C. J.; Tu X. Synthesis, Characterization and Application of Defective Metal-Organic Frameworks: Current Status and Perspectives. J. Mater. Chem. A 2020, 8, 21526–21546. 10.1039/D0TA08009H.

[ref8] Sholl D. S.; Lively R. P. Defects in Metal-Organic Frameworks: Challenge or Opportunity?. J. Phys. Chem. Lett. 2015, 6, 3437–3444. 10.1021/acs.jpclett.5b01135. 26268796

[ref9] Fang Z.; Bueken B.; De Vos D. E.; Fischer R. A. Defect-Engineered Metal-Organic Frameworks. Angew. Chem., Int. Ed. 2015, 54, 7234–7254. 10.1002/anie.201411540. PMC4510710 26036179

[ref10] Dissegna S.; Epp K.; Heinz W. R.; Kieslich G.; Fischer R. A. Defective Metal-Organic Frameworks. Adv. Mater. 2018, 30, 1704501 10.1002/adma.201704501. 29363822

[ref11] Ma N.; Horike S. Metal–Organic Network-Forming Glasses. Chem. Rev. 2022, 122, 4163–4203. 10.1021/acs.chemrev.1c00826. 35044749

[ref12] Gaillac R.; Pullumbi P.; Beyer K. A.; Chapman K. W.; Keen D. A.; Bennett T. D.; Coudert F.-X. Liquid Metal-Organic Frameworks. Nat. Mater. 2017, 16, 1149–1154. 10.1038/nmat4998. 29035353

[ref13] Hou J.; Sapnik A. F.; Bennett T. D. Metal-Organic Framework Gels and Monoliths. Chem. Sci. 2020, 11, 310–323. 10.1039/C9SC04961D. 32153752 PMC7021205

[ref14] Bennett T. D.; Cheetham A. K. Amorphous Metal–Organic Frameworks. Acc. Chem. Res. 2014, 47, 1555–1562. 10.1021/ar5000314. 24707980

[ref15] Bennett T. D.; Horike S. Liquid, Glass and Amorphous Solid States of Coordination Polymers and Metal–Organic Frameworks. Nat. Rev. Mater. 2018, 3, 431–440. 10.1038/s41578-018-0054-3.

[ref16] Tuffnell J. M.; Ashling C. W.; Hou J.; Li S.; Longley L.; Rios Gómez M. L.; Bennett T. D. Novel Metal-Organic Framework Materials: Blends, Liquids, Glasses and Crystal-Glass Composites. Chem. Commun. 2019, 55, 8705–8715. 10.1039/C9CC01468C. 31045184

[ref17] Chapman K. W.; Halder G. J.; Chupas P. J. Pressure-Induced Amorphization and Porosity Modification in a Metal–Organic Framework. J. Am. Chem. Soc. 2009, 131, 17546–17547. 10.1021/ja908415z. 19916507

[ref18] Bennett T. D.; Simoncic P.; Moggach S. A.; Gozzo F.; Macchi P.; Keen D. A.; Tan J.-C.; Cheetham A. K. Reversible Pressure-Induced Amorphization of a Zeolitic Imidazolate Framework (ZIF-4). Chem. Commun. 2011, 47, 7983–7985. 10.1039/c1cc11985k. 21681315

[ref19] Widmer R. N.; Lampronti G. I.; Anzellini S.; Gaillac R.; Farsang S.; Zhou C.; Belenguer A. M.; Wilson C. W.; Palmer H.; Kleppe A. K.; et al. Pressure Promoted Low-Temperature Melting of Metal–Organic Frameworks. Nat. Mater. 2019, 18, 370–376. 10.1038/s41563-019-0317-4. 30886398

[ref20] Bennett T. D.; Goodwin A. L.; Dove M. T.; Keen D. A.; Tucker M. G.; Barney E. R.; Soper A. K.; Bithell E. G.; Tan J.-C.; Cheetham A. K. Structure and Properties of an Amorphous Metal-Organic Framework. Phys. Rev. Lett. 2010, 104, 115503 10.1103/PhysRevLett.104.115503. 20366484

[ref21] Bennett T. D.; Keen D. A.; Tan J.-C.; Barney E. R.; Goodwin A. L.; Cheetham A. K. Thermal Amorphization of Zeolitic Imidazolate Frameworks. Angew. Chem., Int. Ed. 2011, 50, 3067–3071. 10.1002/anie.201007303. 21404398

[ref22] Bennett T. D.; Yue Y.; Li P.; Qiao A.; Tao H.; Greaves N. G.; Richards T.; Lampronti G. I.; Redfern S. A. T.; Blanc F.; et al. Melt-Quenched Glasses of Metal–Organic Frameworks. J. Am. Chem. Soc. 2016, 138, 3484–3492. 10.1021/jacs.5b13220. 26885940

[ref23] Bennett T. D.; Cao S.; Tan J. C.; Keen D. A.; Bithell E. G.; Beldon P. J.; Friscic T.; Cheetham A. K. Facile Mechanosynthesis of Amorphous Zeolitic Imidazolate Frameworks. J. Am. Chem. Soc. 2011, 133, 14546–14549. 10.1021/ja206082s. 21848328

[ref24] Cao S.; Bennett T. D.; Keen D. A.; Goodwin A. L.; Cheetham A. K. Amorphization of the Prototypical Zeolitic Imidazolate Framework ZIF-8 by Ball-Milling. Chem. Commun. 2012, 48, 7805–7807. 10.1039/c2cc33773h. 22760448

[ref25] Bennett T. D.; Saines P. J.; Keen D. A.; Tan J.-C.; Cheetham A. K. Ball-Milling-Induced Amorphization of Zeolitic Imidazolate Frameworks (ZIFs) for the Irreversible Trapping of Iodine. Chem. – Eur. J. 2013, 19, 7049–7055. 10.1002/chem.201300216. 23576441

[ref26] Thorne M. F.; Ríos Gómez M. L.; Bumstead A. M.; Li S.; Bennett T. D. Mechanochemical Synthesis of Mixed Metal, Mixed Linker, Glass-Forming Metal-Organic Frameworks. Green Chem. 2020, 22, 2505–2512. 10.1039/D0GC00546K.

[ref27] Zhou C.; Longley L.; Krajnc A.; Smales G. J.; Qiao A.; Erucar I.; Doherty C. M.; Thornton A. W.; Hill A. J.; Ashling C. W.; et al. Metal-Organic Framework Glasses with Permanent Accessible Porosity. Nat. Commun. 2018, 9, 5042 10.1038/s41467-018-07532-z. 30487589 PMC6262007

[ref28] Frentzel-Beyme L.; Kloß M.; Pallach R.; Salamon S.; Moldenhauer H.; Landers J.; Wende H.; Debus J.; Henke S. Porous Purple Glass − a Cobalt Imidazolate Glass with Accessible Porosity from a Meltable Cobalt Imidazolate Framework. J. Mater. Chem. A 2019, 7, 985–990. 10.1039/C8TA08016J.

[ref29] Hou J.; Ríos Gómez M. L.; Krajnc A.; McCaul A.; Li S.; Bumstead A. M.; Sapnik A. F.; Deng Z.; Lin R.; Chater P. A.; et al. Halogenated Metal–Organic Framework Glasses and Liquids. J. Am. Chem. Soc. 2020, 142, 3880–3890. 10.1021/jacs.9b11639. 31978302

[ref30] Thornton A. W.; Jelfs K. E.; Konstas K.; Doherty C. M.; Hill A. J.; Cheetham A. K.; Bennett T. D. Porosity in Metal–Organic Framework Glasses. Chem. Commun. 2016, 52, 3750–3753. 10.1039/C5CC10072K. 26800518

[ref31] Castillo-Blas C.; Moreno J. M.; Romero-Muñiz I.; Platero-Prats A. E. Applications of Pair Distribution Function Analyses to the Emerging Field of Non-Ideal Metal–Organic Framework Materials. Nanoscale 2020, 12, 15577–15587. 10.1039/D0NR01673J. 32510095

[ref32] Keen D. A. Total Scattering and the Pair Distribution Function in Crystallography. Crystallogr. Rev. 2020, 26, 143–201. 10.1080/0889311X.2020.1797708.

[ref33] Sapnik A. F.; Bechis I.; Collins S. M.; Johnstone D. N.; Divitini G.; Smith A. J.; Chater P. A.; Addicoat M. A.; Johnson T.; Keen D. A.; et al. Mixed Hierarchical Local Structure in a Disordered Metal-Organic Framework. Nat. Commun. 2021, 12, 2062 10.1038/s41467-021-22218-9. 33824324 PMC8024318

[ref34] Groom C. R.; Bruno I. J.; Lightfoot M. P.; Ward S. C. The Cambridge Structural Database. Acta Cryst 2016, 72, 171–179. 10.1107/S2052520616003954 PMC4822653 27048719

[ref35] Baerlocher C.; McCusker L. B. Database of Zeolite Structures, 2017 http://www.iza-structure.org/databases/ (accessed May 1, 2022).

[ref36] Chung Y. G.; Camp J.; Haranczyk M.; Sikora B. J.; Bury W.; Krungleviciute V.; Yildirim T.; Farha O. K.; Sholl D. S.; Snurr R. Q. Computation-Ready, Experimental Metal–Organic Frameworks: A Tool To Enable High-Throughput Screening of Nanoporous Crystals. Chem. Mater. 2014, 26, 6185–6192. 10.1021/cm502594j.

[ref37] Chung Y. G.; Haldoupis E.; Bucior B. J.; Haranczyk M.; Lee S.; Zhang H.; Vogiatzis K. D.; Milisavljevic M.; Ling S.; Camp J. S.; et al. Advances, Updates, and Analytics for the Computation-Ready, Experimental Metal–Organic Framework Database: CoRE MOF 2019. J. Chem. Eng. Data 2019, 64, 5985–5998. 10.1021/acs.jced.9b00835.

[ref38] Thyagarajan R.; Sholl D. S. A Database of Porous Rigid Amorphous Materials. Chem. Mater. 2020, 32, 8020–8033. 10.1021/acs.chemmater.0c03057.

[ref39] Castel N.; Coudert F.-X. Atomistic Models of Amorphous Metal–Organic Frameworks. J. Phys. Chem. C 2022, 126, 6905–6914. 10.1021/acs.jpcc.2c01091.

[ref40] Beake E. O. R.; Dove M. T.; Phillips A. E.; Keen D. A.; Tucker M. G.; Goodwin A. L.; Bennett T. D.; Cheetham A. K. Flexibility of Zeolitic Imidazolate Framework Structures Studied by Neutron Total Scattering and the Reverse Monte Carlo Method. J. Phys.: Condens. Matter 2013, 25, 395403 10.1088/0953-8984/25/39/395403. 24002115

[ref41] Adhikari P.; Xiong M.; Li N.; Zhao X.; Rulis P.; Ching W.-Y. Structure and Electronic Properties of a Continuous Random Network Model of an Amorphous Zeolitic Imidazolate Framework (a-ZIF). J. Phys. Chem. C 2016, 120, 15362–15368. 10.1021/acs.jpcc.6b06337.

[ref42] Wang H.; Li N.; Hu Z.; Bennett T. D.; Zhao X.; Ching W. Y. Structural, Electronic, and Dielectric Properties of a Large Random Network Model of Amorphous Zeolitic Imidazolate Frameworks and Its Analogues. J. Am. Ceram. Soc. 2019, 102, 4602–4611. 10.1111/jace.16308.

[ref43] Yang Y.; Shin Y. K.; Li S.; Bennett T. D.; Van Duin A. C. T.; Mauro J. C. Enabling Computational Design of ZIFs Using ReaxFF. J. Phys. Chem. B 2018, 122, 9616–9624. 10.1021/acs.jpcb.8b08094. 30265536

[ref44] Aldin Mohamed S.; Kim J. Gas Adsorption Enhancement in Partially Amorphized Metal– Organic Frameworks. J. Phys. Chem. C 2021, 125, 4509–4518. 10.1021/acs.jpcc.0c10106.

[ref45] Gaillac R.; Pullumbi P.; Coudert F.-X. Melting of Zeolitic Imidazolate Frameworks with Different Topologies: Insight from First-Principles Molecular Dynamics. J. Phys. Chem. C 2018, 122, 6730–6736. 10.1021/acs.jpcc.8b00385.

[ref46] Gaillac R.; Pullumbi P.; Bennett T. D.; Coudert F.-X. Structure of Metal–Organic Framework Glasses by Ab Initio Molecular Dynamics. Chem. Mater. 2020, 32, 8004–8011. 10.1021/acs.chemmater.0c02950.

[ref47] Addicoat M. A.; Vankova N.; Akter I. F.; Heine T. Extension of the Universal Force Field to Metal–Organic Frameworks. J. Chem. Theory Comput. 2014, 10, 880–891. 10.1021/ct400952t. 26580059

[ref48] Coupry D. E.; Addicoat M. A.; Heine T. Extension of the Universal Force Field for Metal–Organic Frameworks. J. Chem. Theory Comput. 2016, 12, 5215–5225. 10.1021/acs.jctc.6b00664. 27580382

[ref49] Abbott L. J.; Hart K. E.; Colina C. M. Polymatic: A Generalized Simulated Polymerization Algorithm for Amorphous Polymers. Theor. Chem. Acc. 2013, 132, 1334 10.1007/s00214-013-1334-z.

[ref50] Sciortino L.; Alessi A.; Messina F.; Buscarino G.; Gelardi F. M. Structure of the FeBTC Metal–Organic Framework: A Model Based on the Local Environment Study. J. Phys. Chem. C 2015, 119, 7826–7830. 10.1021/acs.jpcc.5b01336.

[ref51] Rivera-Torrente M.; Filez M.; Hardian R.; Reynolds E.; Seoane B.; Coulet M.; Oropeza Palacio F. E.; Hofmann J. P.; Fischer R. A.; Goodwin A. L.; et al. Metal-Organic Frameworks as Catalyst Supports: Influence of Lattice Disorder on Metal Nanoparticle Formation. Chem. – Eur. J. 2018, 24, 7498–7506. 10.1002/chem.201800694. 29709084 PMC6519236

[ref52] Horcajada P.; Surblé S.; Serre C.; Hong D.-Y.; Seo Y.-K.; Chang J.-S.; Grenèche J.-M.; Margiolaki I.; Férey G. Synthesis and Catalytic Properties of MIL-100(Fe), an Iron(III) Carboxylate with Large Pores. Chem. Commun. 2007, 27, 2820–2822. 10.1039/B704325B. 17609787

[ref53] Dhakshinamoorthy A.; Alvaro M.; Garcia H. Commercial Metal–Organic Frameworks as Heterogeneous Catalysts. Chem. Commun. 2012, 48, 11275–11288. 10.1039/c2cc34329k. 23044896

[ref54] Dhakshinamoorthy A.; Alvaro M.; Horcajada P.; Gibson E.; Vishnuvarthan M.; Vimont A.; Grenè J.-M.; Serre C.; Daturi M.; Garcia H. Comparison of Porous Iron Trimesates Basolite F300 and MIL-100(Fe) As Heterogeneous Catalysts for Lewis Acid and Oxidation Reactions: Roles of Structural Defects and Stability. ACS Catal. 2012, 2, 2060–2065. 10.1021/cs300345b.

[ref55] Majano G.; Ingold O.; Yulikov M.; Jeschke G.; Pérez-Ramírez J. Room-Temperature Synthesis of Fe-BTC from Layered Iron Hydroxides: The Influence of Precursor Organisation. CrystEngComm 2013, 15, 9885–9892. 10.1039/C3CE41366G.

[ref56] Czaja A. U.; Trukhan N.; Müiller U. Industrial Applications of Metal-Organic Frameworks. Chem. Soc. Rev. 2009, 38, 1284–1293. 10.1039/b804680h. 19384438

[ref57] Seo Y. K.; Yoon J. W.; Lee J. S.; Lee U.-H.; Hwang Y. K.; Jun C.-H.; Horcajada P.; Serre C.; Chang J. S. Large Scale Fluorine-Free Synthesis of Hierarchically Porous Iron(III) Trimesate MIL-100(Fe) with a Zeolite MTN Topology. Microporous Mesoporous Mater. 2012, 157, 137–145. 10.1016/j.micromeso.2012.02.027.

[ref58] Sanchez-Sanchez M.; De Asua I.; Ruano D.; Diaz K. Direct Synthesis, Structural Features, and Enhanced Catalytic Activity of the Basolite F300-like Semiamorphous Fe-BTC Framework. Cryst. Growth Des. 2015, 15, 4498–4506. 10.1021/acs.cgd.5b00755.

[ref59] Hu X.; Lou X.; Li C.; Ning Y.; Liao Y.; Chen Q.; Mananga S. E.; Shen M.; Hu B. Facile Synthesis of the Basolite F300-like Nanoscale Fe-BTC Framework and Its Lithium Storage Properties. RSC Adv. 2016, 6, 114483–114490. 10.1039/C6RA22738D.

[ref60] Guesh K.; Caiuby C. A. D.; Mayoral A.; Díaz-García M.; Díaz I.; Sanchez-Sanchez M. Sustainable Preparation of MIL-100(Fe) and Its Photocatalytic Behavior in the Degradation of Methyl Orange in Water. Cryst. Growth Des. 2017, 17, 1806–1813. 10.1021/acs.cgd.6b01776.

[ref61] Lohe M. R.; Rose M.; Kaskel S. Metal–Organic Framework (MOF) Aerogels with High Micro- and Macroporosity. Chem. Commun. 2009, 6056–6058. 10.1039/b910175f. 19809642

[ref62] Sapnik A. F.; Ashling C. W.; Macreadie L. K.; Lee S. J.; Johnson T.; Telfer S. G.; Bennett T. D. Gas Adsorption in the Topologically Disordered Fe-BTC Framework. J. Mater. Chem. A 2021, 9, 27019–27027. 10.1039/D1TA08449F.

[ref63] Sapnik A. F.; Johnstone D. N.; Collins S. M.; Divitini G.; Bumstead A. M.; Ashling C. W.; Chater P. A.; Keeble D. S.; Johnson T.; Keen D. A.; et al. Stepwise Collapse of a Giant Pore Metal–Organic Framework. Dalton Trans. 2021, 50, 5011–5022. 10.1039/D1DT00881A. 33877199

[ref64] Jimenez-Solomon M. F.; Song Q.; Jelfs K. E.; Munoz-Ibanez M.; Livingston A. G. Polymer Nanofilms with Enhanced Microporosity by Interfacial Polymerization. Nat. Mater. 2016, 15, 760–767. 10.1038/nmat4638. 27135857

[ref65] Thompson K. A.; Mathias R.; Kim D.; Kim J.; Rangnekar N.; Johnson J. R.; Hoy S. J.; Bechis I.; Tarzia A.; Jelfs K. E.; et al. N-Aryl–Linked Spirocyclic Polymers for Membrane Separations of Complex Hydrocarbon Mixtures. Science 2020, 369, 310–315. 10.1126/science.aba9806. 32675373

[ref66] Vermoortele F.; Ameloot R.; Alaerts L.; Matthessen R.; Carlier B.; Ramos Fernandez E. V.; Gascon J.; Kapteijn F.; De Vos D. E. Tuning the Catalytic Performance of Metal-Organic Frameworks in Fine Chemistry by Active Site Engineering. J. Mater. Chem. 2012, 22, 10313–10321. 10.1039/c2jm16030g.

[ref67] Larsen G. S.; Lin P.; Hart K. E.; Colina C. M. Molecular Simulations of PIM-1-like Polymers of Intrinsic Microporosity. Macromolecules 2011, 44, 6944–6951. 10.1021/ma200345v.

[ref68] Thompson A. P.; Aktulga H. M.; Berger R.; Bolintineanu D. S.; Brown W. M.; Crozier P. S.; In’t Veld P. J.; Kohlmeyer A.; Moore S. G.; Nguyen T. D.; et al. LAMMPS - a Flexible Simulation Tool for Particle-Based Materials Modeling at the Atomic, Meso, and Continuum Scales. Comput. Phys. Commun. 2022, 271, 108171 10.1016/j.cpc.2021.108171.

[ref69] Willems T. F.; Rycroft C. H.; Kazi M.; Meza J. C.; Haranczyk M. Algorithms and Tools for High-Throughput Geometry-Based Analysis of Crystalline Porous Materials. Microporous Mesoporous Mater. 2012, 149, 134–141. 10.1016/j.micromeso.2011.08.020.

[ref70] Tucker M. G.; Keen D. A.; Dove M. T.; Goodwin A. L.; Hui Q. RMCProfile: Reverse Monte Carlo for Polycrystalline Materials. J. Phys. Condens. Matter 2007, 19, 335218 10.1088/0953-8984/19/33/335218. 21694141

[ref71] Robeson L. M. Correlation of Separation Factor versus Permeability for Polymeric Membranes. J. Membr. Sci. 1991, 62, 165–185. 10.1016/0376-7388(91)80060-J.

[ref72] Sapnik A. F.; Bechis I.; Bumstead A. M.; Johnson T.; Chater P. A.; Keen D. A.; Jelfs K. E.; Bennett T. D. Multivariate Analysis of Disorder in Metal-Organic Frameworks. Nat. Commun. 2022, 13, 2173 10.1038/s41467-022-29849-6. 35449202 PMC9023516

[ref73] Farrow C. L.; Billinge S. J. L. Relationship between the Atomic Pair Distribution Function and Small-Angle Scattering: Implications for Modeling of Nanoparticles. Acta Cryst. 2009, 65, 232–239. 10.1107/S0108767309009714. 19349667

[ref74] Keen D. A. A Comparison of Various Commonly Used Correlation Functions for Describing Total Scattering. J. Appl. Crystallogr. 2001, 34, 172–177. 10.1107/S0021889800019993.

[ref75] Canivet J.; Vandichel M.; Farrusseng D. Origin of Highly Active Metal–Organic Framework Catalysts: Defects? Defects!. Dalton Trans. 2016, 45, 4090–4099. 10.1039/C5DT03522H. 26584043

[ref76] Tian J.; Thallapally P. K.; Dalgarno S. J.; McGrail P. B.; Atwood J. L. Amorphous Molecular Organic Solids for Gas Adsorption. Angew. Chem., Int. Ed. 2009, 48, 5492–5495. 10.1002/anie.200900479. 19496094

[ref77] Tian J.; Ma S.; Thallapally P. K.; Fowler D.; McGrail P.; Atwood J. L. Cucurbit[7]Uril: An Amorphous Molecular Material for Highly Selective Carbon Dioxide Uptake. Chem. Commun. 2011, 47, 7626–7628. 10.1039/c1cc12689j. 21660359

[ref78] Jiang S.; Jones J. T. A.; Hasell T.; Blythe C. E.; Adams D. J.; Trewin A.; Cooper A. I. Porous Organic Molecular Solids by Dynamic Covalent Scrambling. Nat. Commun. 2011, 2, 207 10.1038/ncomms1207. 21343925

[ref79] McKeown N. B. Polymers of Intrinsic Microporosity (PIMs). Polymer 2020, 202, 122736 10.1016/j.polymer.2020.122736.

[ref80] Dawson R.; Cooper A. I.; Adams D. J. Nanoporous Organic Polymer Networks. Prog. Polym. Sci. 2012, 37, 530–563. 10.1016/j.progpolymsci.2011.09.002.

[ref81] Kim D.; Liu X.; Lah M. S. Topology Analysis of Metal–Organic Frameworks Based on Metal–Organic Polyhedra as Secondary or Tertiary Building Units. Inorg. Chem. Front. 2015, 2, 336–360. 10.1039/C4QI00236A.

