# Supplementary material for: Modeling the Effect of Defects and Disorder in Amorphous Metal–Organic Frameworks
Source: Chem Mater. 2022 Oct 13;34(20):9042–54. doi: 10.1021/acs.chemmater.2c01528 (PMC9609304; doi:10.1021/acs.chemmater.2c01528)
Supplement: Supplementary file 1 — cm2c01528_si_001.pdf [file cm2c01528_si_001.pdf]

Modeling the effect of defects and disorder in amorphous metal–organic frameworks

SUPPLEMENTARY INFORMATION

Irene Bechis[1], Adam F. Sapnik[2], Andrew Tarzia[1], Emma H. Wolpert[1], Matthew A. Addicoat[3], David A. Keen[4], Thomas D. Bennett[2] and Kim E. Jelfs[1]

[1] Department of Chemistry, Imperial College London, Molecular Sciences Research Hub, White City Campus, London, W12 0BZ, UK.

[2] Department of Materials Science and Metallurgy, University of Cambridge, Cambridge, CB3 0FS, UK.

[3] School of Science and Technology, Nottingham Trent University, Clifton Lane, NG11 8NS Nottingham, UK.

[4] ISIS Neutron and Muon Facility, Rutherford Appleton Laboratory, Harwell Campus, Didcot, Oxfordshire, OX11 0QX, UK

Table of contents

1. Supplementary methodological details 3
1.1. Force Field validation 3
1.2. Amorphous model construction workflow 6
1.2.1. Random packing 6
1.2.2. Polymerization 7
1.2.3. Saturation 9
1.2.4. Annealing 10
1.2.5. Model size effect analysis 13
1.3. Amorphous model analysis 17
1.3.1. Structural analysis 17
1.3.2. Energetic analysis 17
1.3.3. Porosity measurements 18
1.3.4. Pair distribution function calculations 18
1.3.5. Principal component analysis 19
2. Supplementary data 20

1. Supplementary methodological details

1.1. Force Field validation

All the systems studied in this work were treated using the extension of the universal force field (UFF)[1] for metal-organic frameworks UFF4MOF.[2,3] The force field was used without the implementation of partial charges, as it was shown to give a better match with experimental results when used without charges.[4] All the geometry optimization and molecular dynamics simulations involved in the work were performed using the Large-scale Atomic/Molecular Massively Parallel Simulator (LAMMPS)[5] package. The correct implementation of the force field in LAMMPS and its validity in the description of the system studied in this work was initially tested using a small fragment (**Figure S1**) containing the two building blocks of the structure (one trimer and one BTC linker) bonded together. Care was taken in determining the correct UFF4MOF parameters to use in LAMMPS, with the help of available codes (lammps_interface)[4] and the implementation of manual corrections, as already reported in previous work.[6] The UFF4MOF atom types and bond orders used to describe the systems are reported in **Figure S1** and **Table S1**. These values were selected based on the UFF4MOF papers and, in the case of bond orders, comparing to the DFT optimized fragment (in Gaussian16[7] at the B3LYP-D3/6-31G[8,9] level of theory with an effective core potential on the iron atoms, B3LYP-D3/LANL2DZ). Representative LAMMPS input files for the tested fragment can be found at https://github.com/Ibechis/FeBTC_models. Force field energetic terms and geometric features of the optimized test fragment obtained in LAMMPS were compared with the ones obtained from the UFF4MOF implementation in GULP[10] to make sure they were matching, meaning a consistent implementation of the force field in the two codes.

To test the validity of the force field in describing the system, geometrical features such as bonds and angles in the fragment were compared to the DFT optimised fragment. A comparison of the values obtained is reported in **Table S2**. Overall, values obtained with UFF4MOF do not differ by more than 10% from the DFT obtained ones for the considered bonds and angles. To complete the validation, the crystal structure of MIL-100(Fe)[11] was optimized with UFF4MOF in LAMMPS. The asymmetric unit was obtained from the CoRE MOF database[12,13] (refcode: CIGXIA) and then modified in Materials Studio[14] to add two water molecules and one fluorine atom in the terminal coordination position of the iron atoms in each trimer, making the building blocks comparable to the ones used for the amorphous models. The final optimized cubic unit cell contains 12784 atoms and has a lattice parameter of 73.655 Å. This value compares well with the experimentally reported lattice parameter of 73.340 Å (0.44% error). As shown in previous work,[6] the calculated PDF from the model shows good agreement with the experimental PDF of MIL-100(Fe), confirming the validity of the force field used for this study.

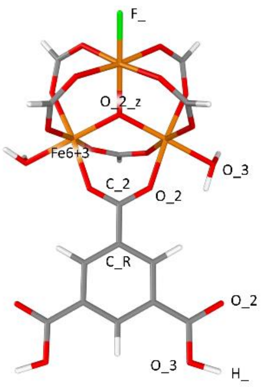

Figure S1 *UFF4MOF atom types used in this work. Carbon is in grey, oxygen in red, iron in orange, fluorine in green and hydrogen in white.*

Atom type1— atom type2	Bond order
O_2 — Fe6+3	0.5
O_2— C_2	1.5
C_2 — C_R	1
C_R — C_R	1.5
C_R — H_	1
O_3 — C_2	1
Fe6+3 — F_	1
Fe6+3 — O_2_z	0.5
Fe6+3 — O_3	0.5
O_3 — H_	1

Table S1 *Selected UFF4MOF bond orders for the atom types involved in the simulated systems.*

Table S2 *Comparison between geometrical features in the test fragment optimized with the UFF4MOF force field in LAMMPS and at B3LYP-D3/6-31G level of theory in Gaussian. For angles we report average values with standard deviations in parenthesis of all the angles in the studied cluster.*

measure	substructure	UFF4MOF (LAMMPS)	DFT (B3LYP-D3/6-31G)
distance	Fe–O_{Carb}	2.05 Å	1.92-1.96 Å
distance	Fe–O_{Oxo}	1.99 Å	1.84-1.96 Å
distance	Fe–O_{water}	2.08 Å	2.02 Å
distance	Fe–F	1.85 Å	1.77 Å
distance	C–O_{trimer}	1.27 Å	1.27-1.29 Å
distance	C_{carb}–C_{ring}	1.48 Å	1.48 Å
distance	C_{ring}–C_{ring}	1.41 Å	1.40 Å
distance	O–H	0.99 Å	0.97-0.98 Å
distance	Fe–Fe	3.45 Å	3.2-3.3
angle	O-C-O	123.47 (0.55)	126.78 (1.21)
angle	Fe-O-C	135.46 (0.73)	128.21 (2.74)
angle	O-Fe-O	91.46 (2.52)	92.20 (3.41)

angle	Fe-O-Fe	120.00 (0.01)	120.00 (0.88)

1.2. Amorphous model construction workflow

We use Polymatic,[15] a polymerisation algorithm developed for the construction of amorphous models of polymers, to build the periodic structure of our amorphous phases of Fe-BTC. **Figure S2** shows a schematic of our workflow, which includes four steps: random packing, polymerization, saturation and annealing.

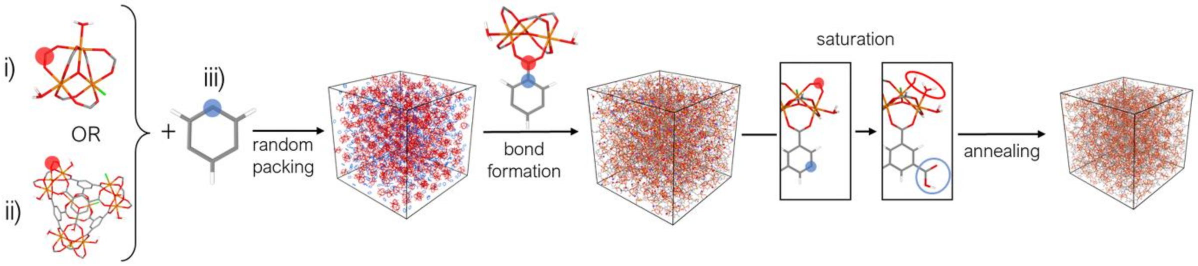

Figure S2 *Schematic of the workflow used to build the structure of the amorphous models. Carbon is in grey, oxygen in red, iron in orange, fluorine in green and hydrogen in white.*

1.2.1. Random packing

Different initial building blocks (trimers, tetrahedra and BTC linkers) were used to tune the degree of order in the models. Their relative quantities in the packing step are reported in **Table S3** and have been decided based on the results of tests on the model size effect reported in **Section 1.2.5**. The size of the models selected represents a good middle ground between computational efficiency and accuracy at all disorder levels**.** In the SRO (short-range order) phases, 400 trimers and 800 BTC linkers were packed in a periodic box with sides of 80 Å in length (initial density of 0.847 g cm^{-3}). In MRO (medium-range order) phases, 100 tetrahedra (one tetrahedron includes four trimers and four BTC linkers already connected in the tetrahedra topology) and 400 BTC linkers were packed in a periodic box with sides of 90 Å in length (initial density of 0.595 g cm^{-3}). In the MIX phases (a middle ground between short-range and medium-range order phases), 50 tetrahedra (which include 200 trimers, 50% of the total number of trimers) and 200 free trimers were packed together with 600 BTC linkers in a periodic box with sides of 85 Å in length (initial density of 0.706 g cm^{-3}). All the models present a one-to-two trimer-to-linkers ratio since trimers are hexacoordinated and BTC linkers are tricoordinate. All the models have 18,800 atoms after the initial random packing, but the total number of atoms of the final models is defined later, in the saturation step. Five independent models for each system were generated by different initial random packings of the building blocks, to ensure good statistical sampling. The packing is performed using the *pack.pl* script provided with Polymatic.

Table S3 *Quantities involved in the initial random packing stage of the structure construction workflow.*

order level	SRO			MIX			MRO		
**defects level**	**<10%**	**20%**	**30%**	**<10%**	**20 %**	**30%**	**<10%**	**20%**	**30%**
packed trimers	400	400	400	200	200	200	0	0	0
packed tetrahedra	0	0	0	50	50	50	100	100	100
packed linkers	800	800	800	600	600	600	400	400	400
packing density (g cm^{-3})	0.847	0.847	0.847	0.706	0.706	0.706	0.595	0.595	0.595

1.2.2. Polymerization

For the polymerization phase, a cut-off distance of 5 Å for the bond formation was used. Bonds are created by the polymerization algorithm between the defined reactive sites, the carbon in the formate unit coordinated to the iron atoms in the trimer (filled red circle in **Figure S2**) and the aromatic carbon in the BTC linker (filled blue circle in **Figure S2**). Following the procedure outlined by Colina and co-workers in reference 15, the whole system was relaxed after the formation of each new bond. Intermediate molecular dynamics (MD) steps in the canonical (NVT) or in the isothermal isobaric (NPT) ensembles were performed in an alternating fashion each time five new bonds were added to the system. These MD cycles are performed to allow building blocks mobility and relaxation of the newly formed clusters of bonded subunits, together with a progressive increase of the initial low density. The NVT MD steps were performed at 1000 K for 10 ps using a timestep of 1 fs. The NPT MD steps were performed at 400 K and 1 bar for 5 ps using a timestep of 1 fs. The Lennard-Jones (LJ) potential was used to model the short-range van der Waals interactions. A cut-off distance for the LJ interactions and the real part of the Ewald summation was set to 15 Å. Constant pressure and temperature were maintained using a Nosé-Hoover thermostat and barostat during MD steps. Opposite fractional charges of 0.5*e* were added to opposite reactive sites to aid the bond formation (and subsequently removed after the bond was formed). The 'intra' flag in polymatic was set equal to 5, to avoid physically unrealistic bonds that connect one linker to the same trimer through multiple bonds. The impact of different polymerisation settings on the final degree of polymerisation, specifically the value of the additional fractional charges on opposite reactive sites, the polymerisation cut-off, the temperature of the NVT runs and the frequency of the NPT runs, have been previously published.[6]

Here, we report the results of those tests, with additional details on the effect of different settings on the final density and porosity of the systems, for those settings that gave at least 60% of polymerisation and allowed for the following saturation and annealing phases. These tests are performed on small models that include 50 trimers and 100 BTC linkers. For each tested setting, results are averaged over three independent models.

We first tested all the settings on a polymerisation cut-off radius of 4 Å, and then raised it to 5 Å on the settings that showed the highest % of polymerisation. We observed that with cut-off values greater than 5 Å, the creation of new bonds sometimes led to distorted structures that caused a failure in the calculation.

We tested the polymerisation with i) no additional charges on opposite reactive sites, ii) with additional charges of 0.3 *e* and iii) 0.5 *e*. We also tested the polymerisation with i) no intermediate MD cycles of NPT type, ii) with intermediate NPT cycles every 10 and iii) every 30 new bonds formed. The temperature and the duration of the NPT steps were set at 400 K and 5 ps respectively, conditions in which we found the simulation to be stable (higher values of temperature or longer simulation times would make the box 'explode' in the initial, more unstable, part of the polymerisation). Intermediate NPT cycles have the goal to gradually decrease the initial low density, in a balanced way, and are particularly important to reach high levels of polymerisation: if the system reaches a higher density too soon in the polymerisation, the mobility of the building blocks is hindered, making bond formation difficult. On the other hand, if the system remains at too low of a density, reactive sites are too far apart to connect. We also tested three different temperatures - 800 K, 1000 K and

1200 K - for the NVT steps that happen every 5 new bonds are formed, unless an NPT cycle is programmed.

Results are reported in **Table S4**. Generally, the presence of additional charges pushes the % of polymerisation up the most. The absence of additional partial charges leads to low % of polymerisation for all tested set ups (entries 1-9). Of these set-ups, only the ones that did not include NPT steps (entries 1-3) reached a % of polymerisation higher than 60%, while the ones including NPT steps (entries 4-9), either every 10 or 30 new bonds, resulted in building blocks floating far away from each other in the first steps of the polymerisation, impeding bond formation. However, when additional partial charges are included (entries 10-29), the presence of NPT runs leads to higher % of polymerisation, with higher frequency of NPT runs (every 10 bonds, entries 13-15 and 22-24) as the best setting. In particular, all the systems that do not include NPT steps during polymerisation show a much lower density and consequently increased porosity. The NVT temperature has a much more subtle effect, pushing the % of polymerisation at slightly higher values when above 1000 K. Finally, a longer cut-off radius (entries 28 and 29) obviously helps to increase the number of bonds formed.
The final density and porosity values are probably partially affected by the small size of the model used for these tests (see **Section 1.2.5** of the SI, on the effect of model size on final properties). Therefore, general trends are more relevant than absolute values.
The polymatic scripts for the polymerization were adapted to work with UFF4MOF. This includes changes necessary to handle the LAMMPS data file for a class I force field and changes in the way improper terms are defined (for an improper involving four atoms *i*, *j*, *k* and *l*, the original Polymatic scripts treat the atom *j* as the central atom, while the *fourier* style used by UFF4MOF in LAMMPS treats atom *i* as the central atom).

Table S4 *Different polymerization setups. Tests were done on a small SRO < 10% system of 2,350 atoms and averaged over three different models. Standard deviations are reported in parenthesis. Entry 29 (reported in green) is the final polymerisation set-up used in this work.* *D_i, D_f and D_{if} are defined in* ***Figure S12****a.*

Entry	Cut-off radius (Å)	Additional Charge (*e*)	frequency of NPT MD	Temperature of NVT MD (K)	Polym (%)	Final density (g cm^{-3})	D_i (Å)	D_f (Å)	D_{if} (Å)
1	4	0.0	never	800	**66.8 (1.1)**	1.01 (0.01)	10.20 (0.23)	4.88 (0.64)	9.94 (0.45)
2	4	0.0	never	1000	**67.8 (1.9)**	1.00 (0.03)	11.50 (1.45)	4.61 (0.81)	10.99 (1.70)
3	4	0.0	never	1200	**69.4 (0.6)**	1.02 (0.06)	11.93 (1.46)	5.13 (1.40)	10.20 (2.17)
4	4	0.0	every 10 bonds	800	**9.5 (1.4)**				
5	4	0.0	every 10 bonds	1000	**9.3 (0.4)**				
6	4	0.0	every 10 bonds	1200	**8.5 (0.7)**				
7	4	0.0	every 30 bonds	800	**35.6 (8.5)**				

8	4	0.0	every 30 bonds	1000	**38.8 (19.6)**				
9	4	0.0	every 30 bonds	1200	**24.3 (5.0)**				
10	4	0.3	never	800	**77.2 (1.3)**	0.98 (0.003)	12.93 (1.62)	6.31 (0.80)	12.42 (2.10)
11	4	0.3	never	1000	**77.8 (2.3)**	1.00 (0.01)	11.92 (0.39)	5.20 (0.67)	11.28 (1.06)
12	4	0.3	never	1200	**78.7 (1.2)**	0.98 (0.03)	10.66 (0.88)	5.21 (0.67)	10.28 (1.38)
13	4	0.3	every 10 bonds	800	**84.1 (1.4)**	1.41 (0.06)	6.71 (0.97)	3.20 (0.41)	6.09 (0.79)
14	4	0.3	every 10 bonds	1000	**85.2 (1.6)**	1.40 (0.02)	6.58 (0.24)	2.91 (0.14)	6.03 (0.28)
15	4	0.3	every 10 bonds	1200	**84.1 (1.5)**	1.42 (0.05)	6.53 (0.52)	2.92 (0.33)	6.06 (0.25)
16	4	0.3	every 30 bonds	800	**83.1 (2.2)**	1.40 (0.04)	6.67 (0.51)	3.02 (0.16)	6.07 (0.32)
17	4	0.3	every 30 bonds	1000	**84.1 (1.2)**	1.38 (0.05)	7.42 (1.04)	2.96 (0.13)	7.31 (1.13)
18	4	0.3	every 30 bonds	1200	**85.1 (0.8)**	1.40 (0.04)	6.59 (0.25)	2.88 (0.31)	6.43 (0.40)
19	4	0.5	never	800	**83.8 (0.5)**	0.96 (0.001	16.03 (2.11)	6.53 (1.66)	15.65 (1.47)
20	4	0.5	never	1000	**84.1 (0.7)**	0.96 (0.03)	15.82 (1.20)	6.85 (1.99)	15.27 (1.38)
21	4	0.5	never	1200	**83.3 (2.2)**	0.97 (0.01)	14.71 (2.73)	8.03 (0.98)	14.67 (2.78)
22	4	0.5	every 10 bonds	800	**88.2 (0.9)**	1.40 (0.05)	7.02 (0.07)	2.98 (0.24)	6.31 (0.70)
23	4	0.5	every 10 bonds	1000	**89.8 (1.3)**	1.42 (0.04)	7.44 (0.31)	3.09 (0.21)	7.31 (0.25)
24	4	0.5	every 10 bonds	1200	**89.6 (0.6)**	1.44 (0.02)	7.32 (0.29)	2.98 (0.29)	6.19 (0.86)
25	4	0.5	every 30 bonds	800	**88.5 (1.6)**	1.36 (0.01)	8.36 (1.93)	3.38 (0.45)	8.13 (2.07)
26	4	0.5	every 30 bonds	1000	**88.5 (2.4)**	1.37 (0.03)	7.98 (0.37)	3.39 (0.68)	7.71 (0.43)
27	4	0.5	every 30 bonds	1200	**87.8 (0.2)**	1.44 (0.01)	7.35 (0.93)	2.84 (0.25)	7.11 (0.94)
28	5	0.3	every 10 bonds	1000	**89.2 (0.5)**	1.48 (0.5)	6.83 (0.40)	2.64 (0.28)	6.31 (0.59)
29	5	0.5	every 10 bonds	1000	**91.6 (2.5)**	91.6 (2.5)	7.33 (1.16)	2.95 (0.31)	7,11 (1.41)

1.2.3. Saturation

In the following step, the additional charges used to help the polymerization were removed from unreacted sites. As the system was treated without the implementation of partial charges, the overall charge of the system remains zero. Unreacted sites were saturated with capping groups. For each missed bond, the unreacted carboxylate group on the trimer (or tetrahedron) was substituted with a water molecule and a hydroxide group on the two adjacent iron atoms left uncoordinated (empty red circle in **Figure S2**), while the unreacted aromatic carbon on the linker was completed with the carboxylic acid functionality (empty blue circle in **Figure S2**). Overall, each missed bond, which we define as a defect in the

structure, causes the addition of two oxygens and four hydrogens to the systems. A complete polymerization, where all reactive sites happen to be within the reaction cut-off of another unreacted site, would give a defect-free structure, but this is unlikely to occur. For this reason, even when the polymerization is allowed to reach completion, a final number of unreacted sites remains present resulting in structures with <10% of defects. Stopping the polymerization at different stages before it reaches its completion allows us to control the amount of bonds formed and, therefore, the number of defects subsequently introduced in the systems during the saturation step. For structures at 30 and 20% of defects, the polymerization was stopped after 1680 and 1920 bonds were formed, respectively (70 and 80% of the total 2400 bonds that could be formed). The saturation is performed through a combination of in-house python scripts, which place capping groups close to the unreacted sites, and the previously described polymerization scripts, used here to connect unreacted sites with capping groups. This is performed in three different steps:

1. First saturation step – iron saturation with H_2O. A python script takes the *final.lmps* file obtained after polymerisation and substitutes the unreacted carboxylate linkers with OH and H_2O groups placed close (but not connected yet) to the two defective iron atoms. Then, the polymerisation scripts from Polymatic are used to create bonds between the first set of iron atoms and the H_2O groups.
2. Second saturation step – iron saturation with OH. A python script takes the file obtained from the first saturation and sets it up for the following polymatic run in which the OH groups get connected to the second set of defective iron atoms.
3. Third saturation step – unreacted linkers saturated with -COOH groups. A python script takes the file obtained from the second saturation and places additional COOH groups close to the unreacted atoms on the linkers. Then, the following polymatic run creates bonds between the unreacted sites on the linker and the carbon of the COOH fragment.

After saturation and before annealing, the structures were fully optimized (using steepest descent and setting the stopping tolerance to 10^{-6} on energy and 10^{-6} Kcal mole^{-1} Angstrom^{-1} on forces).

1.2.4. Annealing

After polymerization and saturation, the structures were annealed through the well-established 21-step MD protocol by Colina and co-workers for the structure generation of amorphous polymers (**Table S5**).[16] The annealing process aims to increase the density of the system, as the initial random packing and polymerization are performed at a lower density to help bond formation, and to allow the system to overcome conformational barriers and relax. We decided to maintain the main framework of the 21-steps in terms of number, duration, order and ensembles of the steps. The 21-step procedure has been derived in such a way that the final density of the obtained system is not dependent on the value of maximum pressure (P_{max}) and maximum temperature (T_{max}) applied to the system. Given that MOFs have a different chemistry than linear and networked polymers, we tested, for one selected system, that this assumption was still true for different values of P_{max} and T_{max}. Additionally, we also ran a longer final equilibration – the 21st step – to check if a longer equilibration was necessary for MOF systems.

Table S6 reports final box size, density and main porosity features for the model 1 of the SRO < 10% system undergoing different annealing set-ups. As expected, lower P_{max} values led to slightly larger box sizes, while the higher P_{max} value led to a slightly smaller final box size. The system annealed for longer shows similar results in terms of final box size, density and porosity compared to the default annealing. Overall, the obtained systems show no significant differences in terms of porosity at different conditions, and the overall standard deviations between all the measured values at different annealing set-ups is smaller than the standard deviation between the five replicas of SRO < 10% (reported as the last column of the table for comparison). Given these results we decided to select the P_{max} and T_{max} values that we routinely adopt for polymeric systems: T_{max} = 1000 K and P_{max} = $5 \cdot 10^4$ bar. T_{final} and P_{final} were set to 300 K and 1 bar, respectively. The equilibration of the structures was checked by plotting the density (**Figure S17-Figure S19**) and the temperature (**Figure S20-Figure S22**) in the last 300 ps of the annealing (NPT simulation at 300 K and 1 bar). Only the final structure for each of the five independent models of each of the nine phases was used for the characterization.

All the obtained structural models are available as xyz, pdb and cif (pdb and cif files include bonding information) at https://github.com/lbechis/FeBTC_models .

Table S5 *The 21-steps molecular dynamics protocol (annealing procedure). Taken from reference 16.*

Step	Ensemble	Conditions	Duration (ps)
1	NVT	T_{Max}	50
2	NVT	T_{Final}	50
3	NPT	T_{Final}, 0.02 (P_{Max})	50
4	NVT	T_{Max}	50
5	NVT	T_{Final}	100
6	NPT	T_{Final}, 0.6 (P_{Max})	50
7	NVT	T_{Max}	50
8	NVT	T_{Final}	100
9	NPT	T_{Final}, P_{Max}	50
10	NVT	T_{Max}	50
11	NVT	T_{Final}	100
12	NPT	T_{Final}, 0.5 (P_{Max})	5
13	NVT	T_{Max}	5
14	NVT	T_{Final}	10
15	NPT	$T_{Final,}$ 0.1 (P_{Max})	5
16	NVT	T_{Max}	5
17	NVT	T_{Final}	10
18	NPT	T_{Final}, 0.01 (P_{Max})	5
19	NVT	T_{Max}	5
20	NVT	T_{Final}	10
21	NPT	T_{Final}, P_{Final}	800

***Table S6** Annealing settings (shaded in blue) tested for the model 1 of SRO < 10% defects system and final structural characteristics (shaded in white) of the annealed model. Last two columns report the standard deviation between values obtained by different annealing settings and the standard deviation between the five models of SRO < 10% defects. D_i, D_f and D_{if} are defined in **Figure S12**a. ASA = accessible surface area, NASA = non-accessible surface area.*

	**default**	**longer**	**Lower P_{max}**	**Lower P_{max}**	**Higher P_{max}**	**Lower T_{max}**	**Lower T_{max}**	**Higher T_{max}**	**Higher T_{max}**	**St. dev.**	**SRO <10% st. dev.**
21st step (ps)	800	1600	800	800	800	800	800	800	800	-	**-**
P_{max} (bar)	$5\cdot10^4$	$5\cdot10^4$	$1\cdot10^3$	$5\cdot10^3$	$5\cdot10^5$	$5\cdot10^4$	$5\cdot10^4$	$5\cdot10^4$	$5\cdot10^4$	-	**-**
T_{max} (K)	1000	1000	1000	1000	1000	600	800	1200	1400	-	**-**
Final box size (Å)	66.77	66.78	66.82	66.88	66.72	66.80	66.85	66.83	66.83	0.05	**0.32**
Final density (g cm^{-3})	1.49	1.49	1.49	1.49	1.50	1.49	1.49	1.49	1.49	0.003	**0.02**
Final skeletal density (g cm^{-3})	1.56	1.56	1.56	1.55	1.56	1.56	1.56	1.56	1.56	0.002	**0.01**
D_i (Å)	8.09	8.74	8.76	8.74	8.76	8.91	8.82	8.93	9.09	0.28	**0.90**
D_f (Å)	2.87	2.73	2.85	2.96	2.79	2.85	2.80	2.82	3.00	0.08	**0.11**
D_{if} (Å)	7.43	6.04	8.75	8.72	7.28	8.74	8.00	7.50	9.00	0.97	**1.08**
ASA (m^2 g^{-1}, D = 2.4 Å)	852	847	853	868	842	870	868	852	861	10	**56**
NASA (m^2 g^{-1}, D = 2.4 Å)	76	81	80	78	77	63	77	87	76	6	**15**

1.2.5. Model size effect analysis

To address the effect of model size, we built three additional sets of models of different sizes, including, respectively 40, 200 and 800 trimers and we compared them with the models presented in the main papers, which contain 400 trimers. We built these sets of models both with free trimers (SRO-type models) and with the trimers pre-assembled in tetrahedra (MRO-type models), the two extremes on the disorder scale. We allowed the polymerisation to run until no further reactive sites could be found within the set 5 Å cut-off radius (i.e., we are studying the effect of the box size on the models at < 10% of defect content). For each model we built three replicas starting from three initial random packings.

Table S7 reports the specifics of the model construction (trimer/tetrahedra/linker ratio and initial density/box size) and the characteristics of the final structure after annealing, i.e. density, final box size and porosity. As for the rest of the manuscript, the porosity analysis reported is geometrical and performed by keeping the framework rigid. Standard deviations over the three packings (five for the set including 400 trimers, the one presented in the main paper) are reported in parentheses. These features are also plotted as a function of box size in **Figure S3-Figure S4**. Generally, the results show that box size effect is small within the accuracy of the model, plateauing for most measured properties at the 400-trimer containing models.

Bigger models are better in terms of ability to sample different atomic environments and pores in the structure, but the computational cost of the simulations involved in their construction increases quickly with the dimension of the model, imposing the necessity to find an acceptable trade-off between accuracy and computational cost. The increased computational cost derives from calculations of bonded terms (scale with N atoms in the system) and pairwise interactions (scale with N^2) between a higher number of atoms, but also because of how the polymerisation phase is performed. Indeed, a higher number of building blocks requires a higher number of bonds to be formed, which in turn requires more intermediate MD steps to be run every 5 new bonds formed. For this reason, **Table S7** also reports a rough estimate of the time needed to perform the polymerisation phase for each system. Calculations were performed in the HPC systems at Imperial College London (RCS facilities). Compute resources required for these calculations were 48 cores per node using 124Gb RAM memory. MRO models are faster, because even if they contain the same number of atoms as the respective SRO models, they require less bonds to be formed, as the trimers are pre-assembled in tetrahedra.
The selected box size (400 trimers) allows a good exploration of all the phases at different disorder levels and represents a good middle ground between computational efficiency and accuracy.

Looking at the obtained results in more detail, the final density of the models tends to increase by increasing the system size, both for the SRO and MRO systems. The standard deviation of the density between replicas of the same system also decreases with size, although it is particularly higher only in the smaller 40-trimers models. The effect of increasing box size on porosity is generally less marked for SRO models compared to MRO models. For SRO models (**Figure S3**), increasing the model size decreases the standard deviation in the surface area values and shifts the PSD to lower values. D_f value and the surface area decrease, but the pore dimension (D_i and D_{if} values) increase. For MRO models

(**Figure S4**)**,** a stronger box size effect is especially visible in the PSD, with the bigger 400-trimers and 800-trimers boxes showing close results. These two box sizes also show similar values of surface areas. A reduction in the standard deviation with increasing box size is present in all the properties reported in **Figure S4**a-c, with the general tendency of bigger models to become slightly less porous, in line with a higher density value (see surface area and *D* values)**.**

Additionally, we calculated the PDF of the models, to address any box size effect on this feature (**Figure S5**). The PDFs for different box sizes are very similar at low *r*. The box size effect becomes more visible at *r* >7 Å, where bigger models present fewer individual features but a broader overall oscillation. The oscillating background in the PDF rises from the very low-Q peak in the structure factor produced by big models (similar to the low-Q feature arising from the tetrahedral units highlighted in **Figure S15**) and is not present in experimental PDFs. Thus, too big models pose a problem when comparing with experimental results, as the additional (much longer) length scales are not really present in the PDF experimental data, as experimental diffraction measurements do not go to low enough Q.

Table S7 *Box size effect study on SRO and MRO models. Results are averaged over three models with standard deviation in parenthesis (except the SRO models with trimer/tetrahedra/linker ratio of 400/0/800 and the MRO model with 0/100/400 ratio, which are the structures presented in the manuscript and are averaged over 5 models).* D_i*,* D_f *and* D_{if} *are defined in* ***Figure S12****a. ASA = accessible surface area, NASA = non-accessible surface area.*

Model type	SRO	SRO	**SRO**	SRO	MRO	MRO	**MRO**	MRO
trimer/tetrahedra/linker ratio	40/0/80	200/0/400	**400/0/800**	800/0/1600	0/10/40	0/50/200	**0/100/400**	0/200/800
Initial size (Å)	40	65	**80**	105	45	75	**90**	120
Initial density (g cm^{-3})	0.677	0.789	**0.847**	0.749	0.476	0.514	**0.595**	0.502
% polymerization	92.0 (0.2)	92.8 (0.0)	**92.1 (0.1)**	93.5 (0.4)	89.2 (1.9)	91.8 (0.6)	**91.5 (0.3)**	93.3 (0.4)
Missing bonds	19 (1)	87 (1)	**189 (24)**	312 (20)	26 (5)	99 (7)	**205 (6)**	323 (19)
Final n of atoms	1996 (3)	9919 (4)	**19932 (142)**	39474 (123)	2036 (27)	9996 (43)	**20034 (32)**	39596 (195)
Final size	31.51 (0.36)	53.28 (0.17)	**66.77 (0.32)**	83.92 (0.29)	36.21 (2.12)	57.91 (0.60)	**71.36 (0.29)**	88.87 (0.55)
Final bulk density (g cm^{-3})	1.42 (0.05)	1.47 (0.01)	**1.49 (0.02)**	1.50 (0.01)	0.96 (0.15)	1.15 (0.03)	**1.23 (0.02)**	1.26 (0.02)
Final skeletal density (g cm^{-3})	1.52 (0.03)	1.54 (0.01)	**1.57 (0.01)**	1.56 (0.01)	1.36 (0.03)	1.41 (0.01)	**1.45 (0.01)**	1.46 (0.01)
D_i (Å)	7.29 (0.32)	7.98 (1.00)	**8.03 (0.90)**	8.70 (0.23)	16.66 (5.35)	13.95 (2.20)	**14.96 (1.67)**	15.62 (0.81)
D_f (Å)	3.00 (0.04)	2.92 (0.16)	**2.94 (0.11)**	2.81 (0.02)	7.77 (2.24)	5.78 (0.84)	**4.92 (0.66)**	4.09 (0.17)
D_{if} (Å)	6.66 (0.44)	7.25 (1.15)	**7.62 (1.08)**	8.08 (1.24)	16.06 (5.26)	13.39 (2.68)	**13.43 (2.37)**	13.04 (0.30)
ASA (m^2 g^{-1}, r = 1.2 Å)	1071 (211)	957 (31)	**873 (56)**	861 (47)	1914 (152)	1621 (42)	**1411 (57)**	1339 (34)
NASA (m^2 g^{-1}, r = 1.2 Å)	45 (62)	46 (15)	**58 (15)**	66 (13)	106 (7)	105 (2)	**105 (4)**	108 (5)
Time for polymerisation (1 node, 48 cores)	~40 min	~11 hours	**~3 days**	~ 3 weeks	~20 min	~3 hours	**~15 hours**	~ 4 days

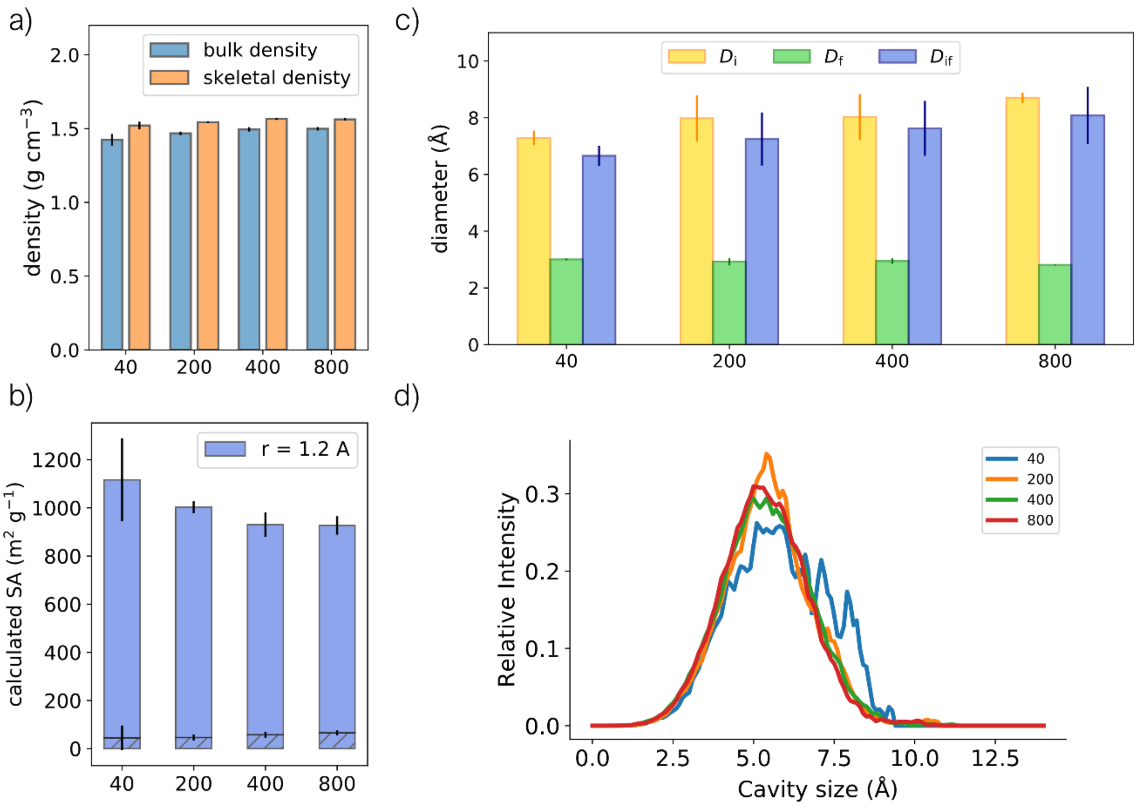

Figure S3 *Box size effect on MRO at < 10% defect models. The four sets of models at different size are labelled by the number of trimers contained in the structure (40, 200, 400, 800). The models presented in the manuscript are the ones including 400 trimers. a) Final bulk and skeletal density. b) Accessible (plain) and non-accessible (hatched) surface area for a probe of 1.2 Å in radius (diameter = 2.4 Å). c) Porosity features of the models,* D_i, D_f *and* D_{if}, *as defined in* ***Figure S12****a. d) PSD (probe diameter = 0.2 Å).*

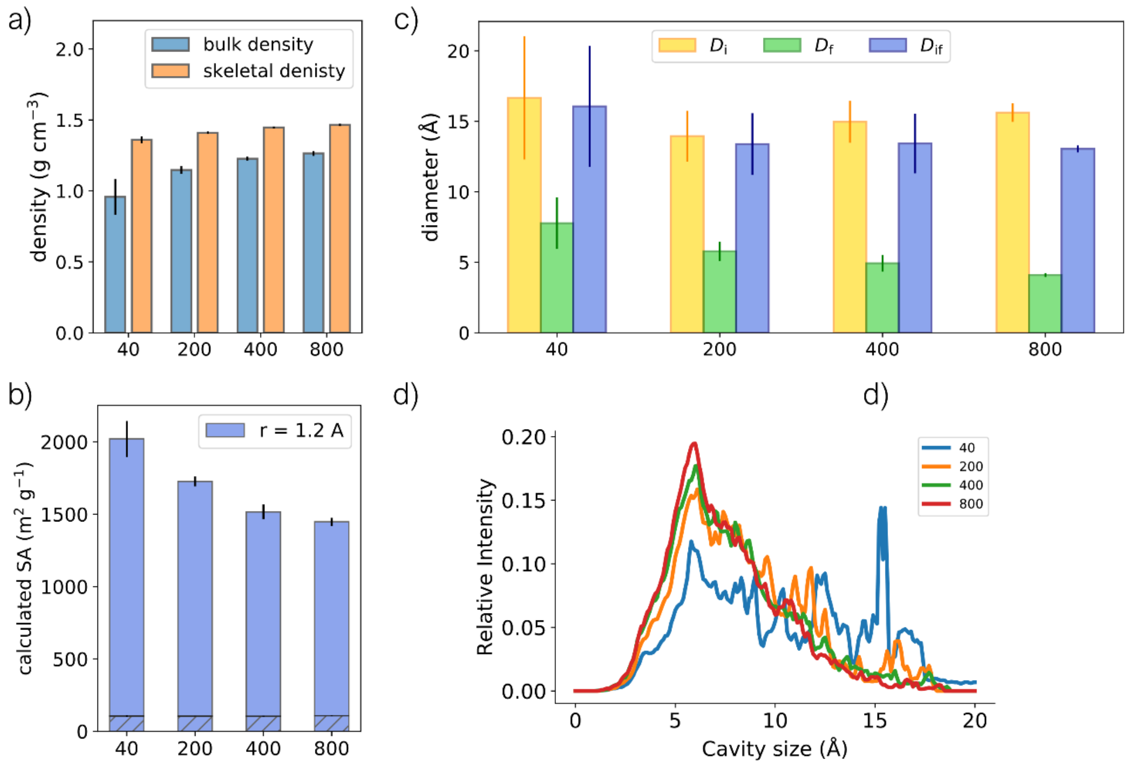

Figure S4 *Box size effect on MRO at < 10% defect models. The four sets of models at different size are labelled by the number of trimers contained in the structure (40, 200, 400, 800, which means 10, 50, 100 and 200 tetrahedra, respectively). The models presented in the manuscript are the ones including 400 trimers. a) Final bulk and skeletal density. b) Accessible (plain) and non-accessible (hatched) surface area for a probe of 1.2 Å in radius (diameter = 2.4 Å). c) Porosity features of the models,* D_i*,* D_f *and* D_{if}*, as defined in* ***Figure S12****a. d) PSD (probe diameter = 0.2 Å).*

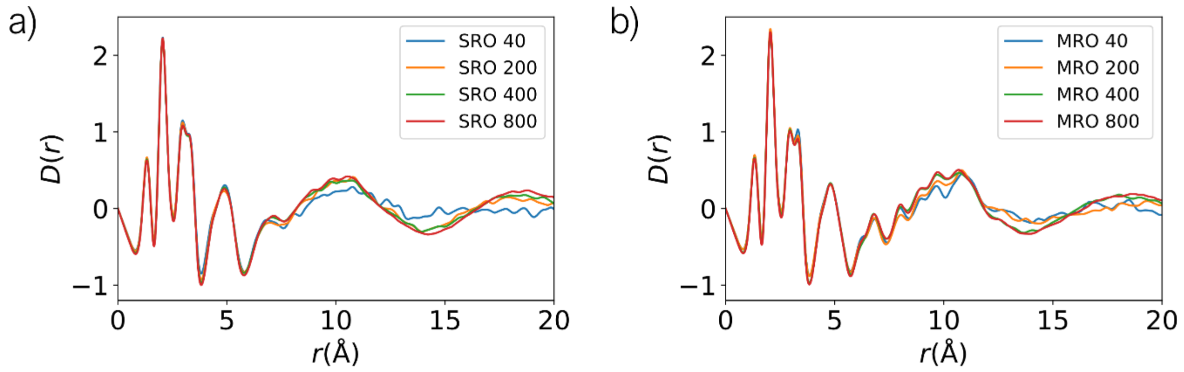

Figure S5 *Box size effect on the PDF for a) one representative model for the SRO < 10% system and b) one representative model for the MRO < 10 %.*

1.3. Amorphous model analysis

1.3.1. Structural analysis

Radial distribution functions (RDFs, **Figure S7**) involving the iron atoms and the trimer centers (atom type = O_2_z) were calculated using OVITO[17] (version 2.9.0) with 150 histogram bins and a cut-off distance of 30 Å. Centroid-centroid distances and interplanar angles for pairs of BTC linkers (**Figure S8**) were obtained with an in-house python script that extracts the centroid of the benzene ring of the BTC linker using the coordinates of three of the carbon atoms in the ring. The script then calculates the distance between centroids and the interplanar angles between the plane passing through the three benzene carbons for pairs of linkers whose centroids fall within a 7 Å cut-off. Values for structural features like bonds, angles and dihedrals values (**Figure S10**) were calculated directly from LAMMPS using the LAMMPS commands:

```
compute 1 all property/local dtype datom1 datom2 datom3 datom4
compute 2 all dihedral/local phi

compute 3 all property/local atype aatom1 aatom2 aatom3
compute 4 all angle/local theta

compute 5 all property/local btype batom1 batom2
compute 6 all bond/local dist

dump 1 all local 1 dihedrals.traj c_1[1] c_1[2] c_1[3] c_1[4] c_1[5] c_2
dump 2 all local 1 angles.traj c_3[1] c_3[2] c_3[3] c_3[4] c_4
dump 3 all local 1 bonds.traj c_5[1] c_5[2] c_5[3] c_6
```

To analyse the distortion of oxo-trimer centers, iron octahedral centers and phenyl linkers in the models, we calculated the trigonal planar order parameter, octahedral order parameter[18] and deviation from planarity, respectively of those local environments. The deviation from planarity is defined as the sum of the distance of all six carbons from the plane of best fit through them. Order parameters were calculated using pymatgen[19] (version 2022.0.16). These measures quantify how much the substructure fits an octahedral, trigonal planar and a planar geometry, respectively (**Figure S11**). The LAMMPS data files were parsed using MDAnalysis (version 2.0.0)[20,21] and NetworkX[22] (version 2.6.3). To calculate planarity, each model is converted to a NetworkX graph containing only the atoms and bonds associated with the target local geometry (*e.g.,* six "C_R" carbon atoms). To calculate the order parameters, all target central atoms (either the "O_2_z" or the "Fe6+3" atom types) and their connected atoms (either 3 or 6 for the trigonal planar or octahedral order parameter, respectively) are converted into pymatgen structures, on which the order parameters are calculated.

1.3.2. Energetic analysis

The average energy per trimer values used in **Figure 5**a and **Figure S6** were taken from the global potential energy value of the last timestep of the annealing in LAMMPS (sum of *evdwl, ecoul*, *ebond*, *eangle*, *edihed*, *eimp* terms).

The force field energy of each individual atom used for the plot in **Figure 5**b and **Figure S9** was obtained from the last timestep of the annealing using the *pe_atom* LAMMPS function with the following LAMMPS commands:

```
compute pe all pe/atom
compute pair all pe/atom pair
compute bond all pe/atom bond
compute angle all pe/atom angle
compute dihedral all pe/atom dihedral
compute improper all pe/atom improper
dump 1 all custom 1000 energy.trj id type x y z c_pe c_bond c_angle c_pair c_dihedral c_improper
```

and plotted using OVITO. The *pe_atom* function equally partitions the energy contribution of a group of atoms between the atoms in the group (e.g., a 50/50 split for two atoms in a bond, a 25/25/25/25 split for the four atoms in a torsion term etc).

1.3.3. Porosity measurements

The geometrical porosity features of the rigid models (pore size distribution (PSD), surface area and pore volume) were calculated using Zeo++.[23] The probe used is always spherical and when it has the same diameter of atoms and molecules the results can give us an insight into which possible guests could pass through the model based on their size. However, this approach does not consider the possible interactions of the guest with the host material or guest shapes different from a sphere. The accessible and non-accessible surface area values and pore volumes in **Table S9** were calculated with a probe diameter of 2.40 Å and 3.64 Å (kinetic diameter of N_2)[24] using 7000 Monte Carlo (MC) samples per atom for the surface area and 70000 MC samples per unit cell for the volume. The accessible surface area as a function of probe size in **Figure S12**b was calculated with probe diameters from 0.40 to 6.00 Å and 5000 MC samples per atom. The visual representation of accessible and non-accessible surface area was obtained using 1500 MC samples per atom and a probe diameter of 2.60 Å. Pore size distributions were calculated using 70000 MC samples per cell and a small probe diameter of 0.20 Å. All the calculations were performed using the high accuracy flag.

1.3.4. Pair distribution function calculations

Total pair distribution functions were calculated using the RMCProfile[25] software package. Firstly, the partial pair distribution functions, $g_{ij}(r)$, were obtained and the Fourier transform calculated to yield the partial structure factor, $A_{ij}(Q)$. The $A_{ij}(Q)$ functions were multiplied by their respective Q-dependent X-ray weighting coefficients and summed to obtain the total structure factor, $F(Q)$.[26] The Fourier transform of $F(Q)$ was calculated to give to total pair distribution function, $G(r)$. We make use of the $D(r)$ form of the PDF, $D(r) = 4\pi r \rho G(r)$ to accentuate correlations at high-r. We report the PDFs for a representative model of each phase type, after assessing the low deviation between the five different models for the same system (**Figure S13**).

1.3.5. Principal component analysis

Principal component analysis (PCA) was used to extract the latent variables of the PDF data. PCA is a dimensionality reduction technique that projects the original data onto an orthogonal basis whose directions are found by simultaneously maximizing the variance of the data and minimising the reconstruction error. PCA was carried out as described in our previous work.[27] Briefly, PCA was carried out using the correlation matrix option within the Origin Pro software package. Principal components were retained such that (i) a minimum threshold of 99% of the statistical variance of the data was captured and (ii) at least two components were retained.

The statistical variance of the first three components (PC1, PC2 and PC3) obtained from PCA of the PDF series studied in this work is reported in **Table S10**. The second component (PC2), termed the distortion component, describes how a series of PDFs deviates from the statistical average of the dataset. The third components (PC3) contained high-frequency noise and were discarded based on the criteria outlined above. Distortion components (PC2) for the series of PDFs studied in this work are reported in **Figure S16**.

2. Supplementary data

Table S8 *Additional information on the amorphous structure construction workflow, including the average number of unreacted sites after polymerization, the average final number of atoms after saturation and the average final box size obtained after annealing for each of the nine phases studied. Standard deviations between the five independent models are reported in parentheses.*

**order level**	**SRO**			**MIX**			**MRO**		
**defects level**	**<10%**	**20%**	**30%**	**<10%**	**20%**	**30%**	**<10%**	**20%**	**30%**
**unreacted sites**	189 (24)	479 (1)	719 (1)	187 (11)	480	720	205 (6)	480	720
**atoms after annealing**	19932 (142)	21676 (8)	23116 (8)	19921 (69)	21680	23120	20034 (32)	21680	23120
**final box size (Å)**	66.77 (0.32)	68.67 (0.37)	69.86 (0.38)	68.09 (0.35)	70.17 (0.09)	71.33 (0.09)	71.36 (0.29)	73.73 (0.28)	74.43 (0.45)

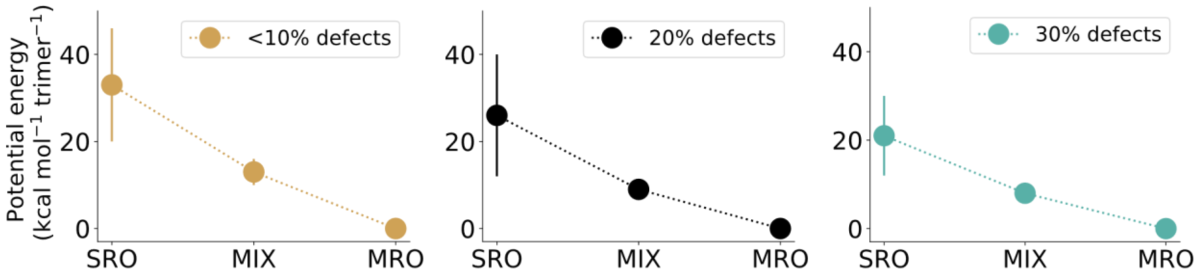

Figure S6 *Average potential energy per trimer in each of the nine studied phases. The error bars refer to the standard deviation over the five individual models for each phase. Energies for systems at the same level of defects are quantitatively comparable because they contain the same numbers and types of atoms and bonds (except for the <10% defect level, in which there is a small deviation because the polymerisation is allowed to run until no further reactive sites within the established cut-off are found). SRO phases at all explored level of defects show a much higher standard deviation across the five models compared to MIX and MRO phases, showing a higher variability in the energy depending on the initial packing.*

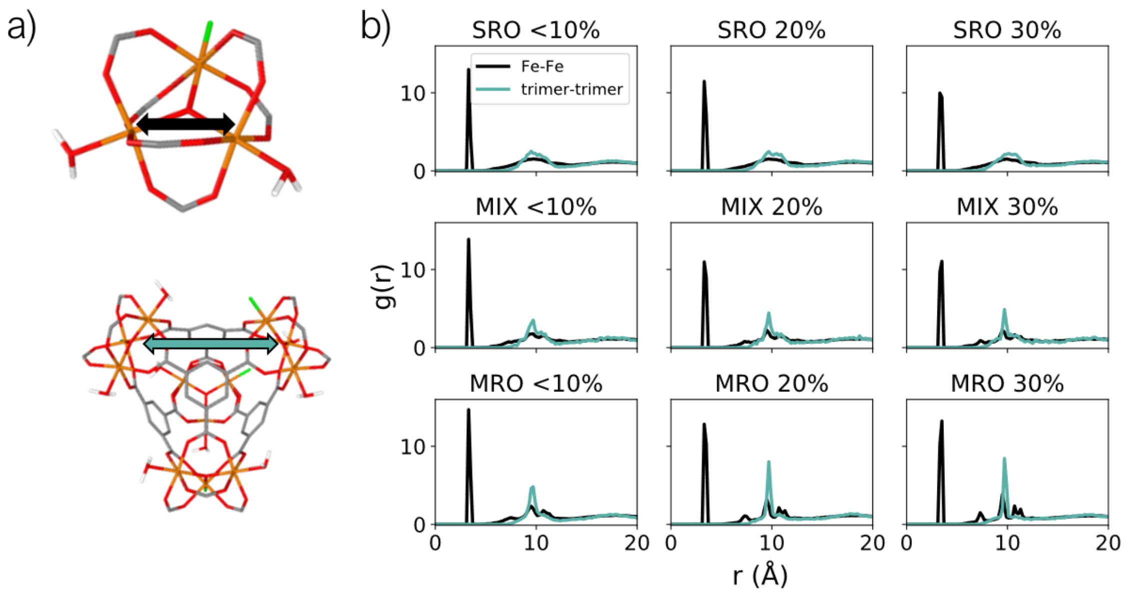

Figure S7 *a) Representation of the (top) Fe-Fe distance and (bottom) trimer-trimer distance. b) Plot of the RDF for trimer centers and Fe atoms in the nine phases. Defined peaks at around 10 Å for both features highlight the presence of tetrahedra in the structure. Narrower and more intense peaks at higher level of defects for MIX and MRO phases indicate a more regular arrangements for tetrahedra when less strained by cross-linking.*

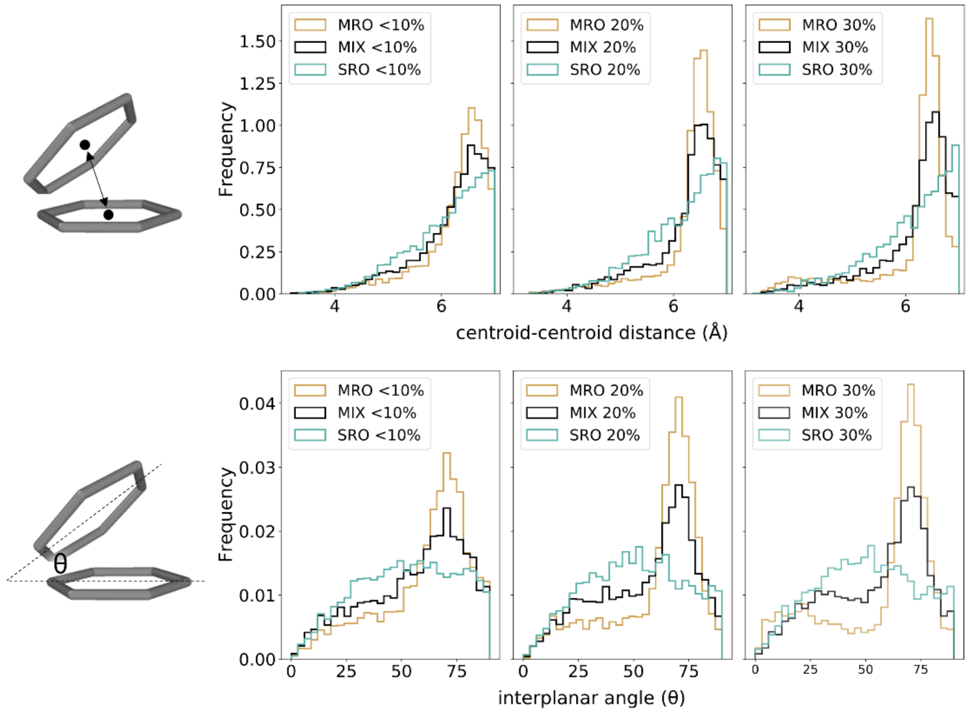

Figure S8 *(top) Centroid-centroid distance and (bottom) interplanar angle distributions for pairs of BTC linkers within a cutoff of 7.0 Å for the models at different level of order with < 10 % defects (left), 20 % defects (middle) and 30 % defects (right). The distributions show that the more ordered architecture of the tetrahedron produces distributions centered around specific values (around 6.5 Å for the centroid-centroid distance and 70° for the interplanar angle) in the MIX and MRO phases compared to the SRO phases, where linkers are oriented in a more disordered fashion. For MIX and MRO phases, higher level of defects show a distributions with narrower and more intense peaks, sign of tetrahedra adopting a more uniform and less distorted structure because of decreased strain.*

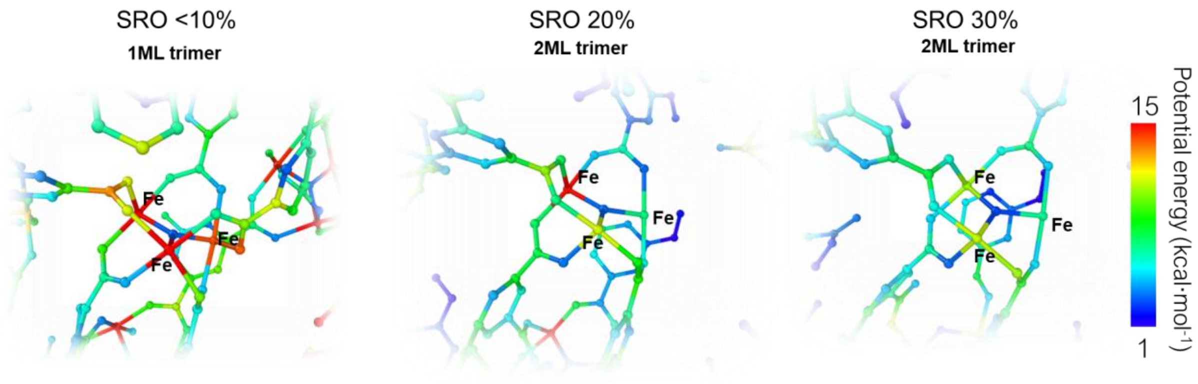

Figure S9 *Representative image of a trimer in one of the SRO models at different levels of defects: (left) <10%, (middle) 20% and (right) 30%. The atoms are color-coded according to their UFF4MOF potential energy. Iron atoms have the highest energy in less defective structures (<10%), while they are more relaxed when one or more linkers are missing and are substituted by 'free' (i.e., not interconnected) OH/H_2O groups in more defective models (20, 30% of defects). This confirms that defects reduce the strain experienced by the building blocks of the structure when cross-linked. The image shows a slice of the model with hydrogen atoms, groups in terminal positions (H_2O and F) and capping groups for defects (H_2O, OH, COOH) removed for clarity and to highlight the effect of defects on the iron atoms.*

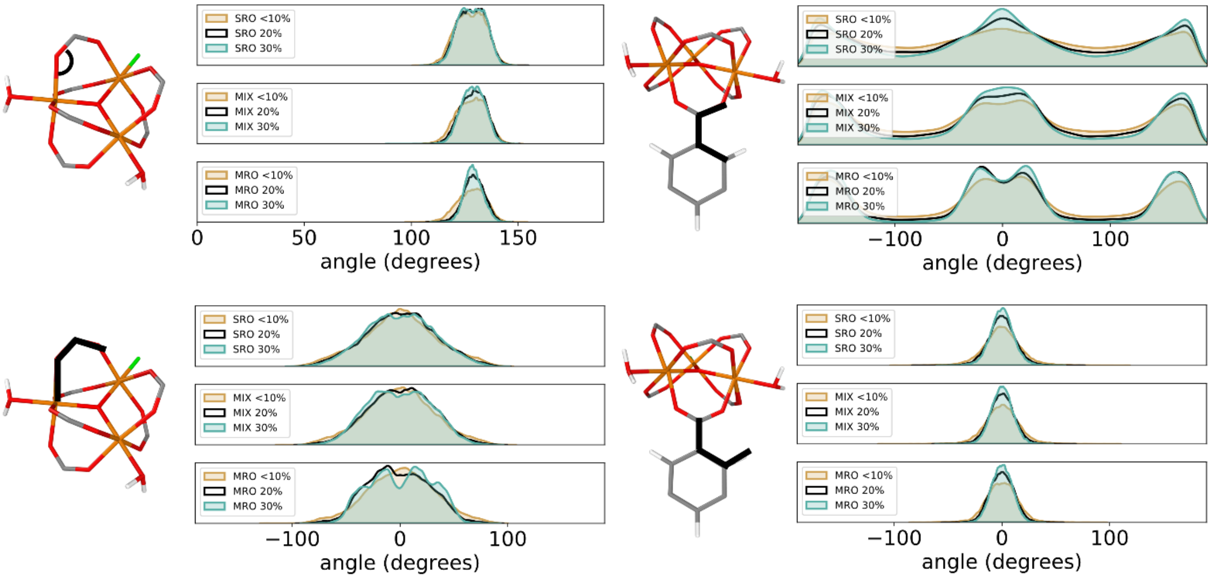

Figure S10 *Distributions of bond and dihedral angles (highlighted in black) in the amorphous models. Models at the same level of order (SRO on top, MIX in the middle and MRO on the bottom) but different levels of defects are shown together. Models at higher level of defects show narrower distribution of values for these features, which are the most involved in the bond formed by the polymerization. This is an additional confirmation of the formation of less distorted and more homogenous structures at higher levels of defects. Carbon is in grey, oxygen in red, iron in orange, fluorine in green and hydrogen in white.*

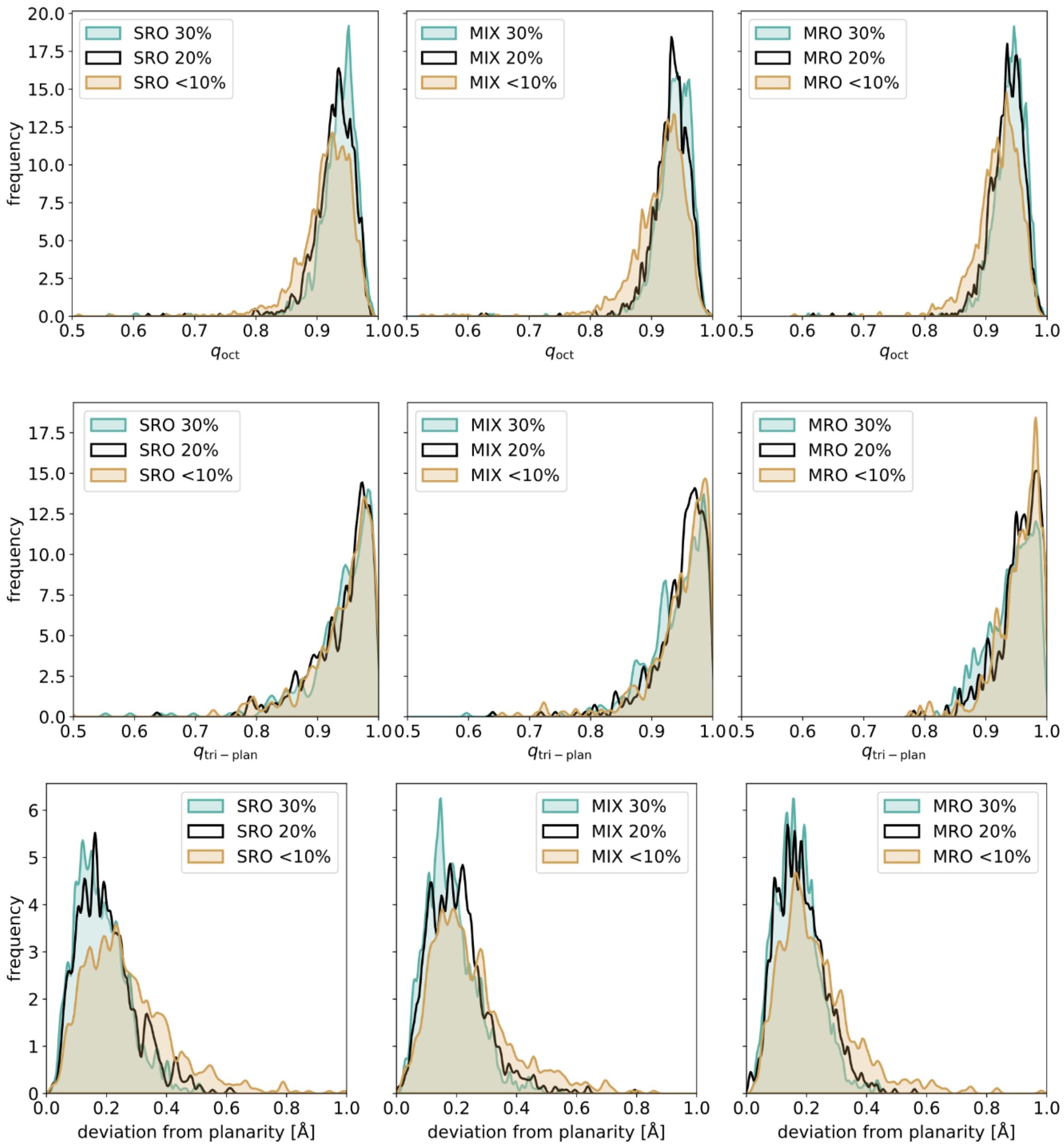

***Figure S11** Octahedral order-parameter (top), trigonal planar order parameter (middle) and deviation from planarity of benzene rings (bottom), extracted from the final timestep of model 1 of all nine amorphous phases. This metric does not highlight significant variations for trimers and iron octahedra as a function of the degree of disorder or defects, but it shows slightly more planar BTC linkers in defective systems, in agreement with the decreased strain of these structures.*

Table S9 *Calculated porosity metrics of the nine studied amorphous phases. Only average values over the five independent models are reported, standard deviations are in parenthesis. Accessible surface areas (ASA) and non-accessible surface areas (NASA) are calculated using probe diameters of 2.40 Å and 3.64 Å (equivalent to the kinetic diameter of a* N_2 *molecule). Available volume (Av. vol) and non-available volume (Nav. vol) are reported for a probe diameter of 2.40 Å.*

**order level**		**SRO**			**MIX**			**MRO**		
**defects level**		**<10%**	**20%**	**30%**	**<10%**	**20%**	**30%**	**<10%**	**20%**	**30%**
D_i (Å)		8.03 (0.90)	7.59 (0.81)	7.24 (0.46)	9.15 (0.56)	9.65 (0.72)	9.42 (0.45)	14.96 (1.67)	14.70 (0.96)	13.10 (1.54)
D_f (Å)		2.94 (0.11)	2.75 (0.20)	2.71 (0.13)	3.19 (0.10)	3.08 (0.11)	3.00 (0.16)	4.92 (0.66)	4.67 (0.75)	4.50 (0.44)
D_{if} (Å)		7.62 (1.08)	6.94 (1.07)	6.25 (0.63)	8.21 (0.65)	8.37 (1.05)	8.84 (1.05)	13.43 (2.37)	13.55 (1.44)	12.25 (1.69)
	probe diameter (Å)									
ASA (m^2 g^{-1})	2.40	873 (56)	889 (92)	794 (138)	1071 (65)	1125 (23)	1082 (24)	1411 (57)	1562 (24)	1477 (71)
NASA (m^2 g^{-1})	2.40	58 (15)	75 (18)	133 (56)	75 (7)	86 (12)	87 (9)	105 (4)	106 (1)	105 (5)
Tot SA (m^2 g^{-1})	2.40	930 (45)	963 (77)	927 (84)	1145 (59)	1211 (16)	1169 (21)	1516 (55)	1668 (24)	1582 (66)
Av. vol (cm^3 g^{-1})	2.40	0.040 (0.006)	0.039 (0.006)	0.032 (0.008)	0.060 (0.005)	0.060 (0.003)	0.055 (0.003)	0.137 (0.008)	0.148 (0.009)	0.129 (0.012)
Nav. vol (cm^3 g^{-1})	2.40	0.001 (0.000)	0.002 (0.001)	0.004 (0.002)	0.002 (0.000)	0.003 (0.000)	0.003 (0.000)	0.004 (0.000)	0.004 (0.000)	0.004 (0.000)
TOT vol (cm^3 g^{-1})	2.40	0.042 (0.005)	0.040 (0.006)	0.036 (0.011)	0.062 (0.005)	0.063 (0.002)	0.061 (0.007)	0.141 (0.008)	0.152 (0.009)	0.133 (0.012)
ASA (m^2 g^{-1})	3.64 (N_2)	-	-	-	-	-	-	724 (77)	804 (45)	693 (56)
NASA (m^2 g^{-1})	3.64 (N_2)	243 (36)	227 (46)	199 (53)	392 (35)	385 (17)	346 (25)	78 (28)	70 (8)	94 (27)
Tot SA (m^2 g^{-1})	3.64 (N_2)	243 (36)	227 (46)	199 (53)	395 (35)	385 (17)	346 (25)	803 (51)	874 (38)	787 (50)

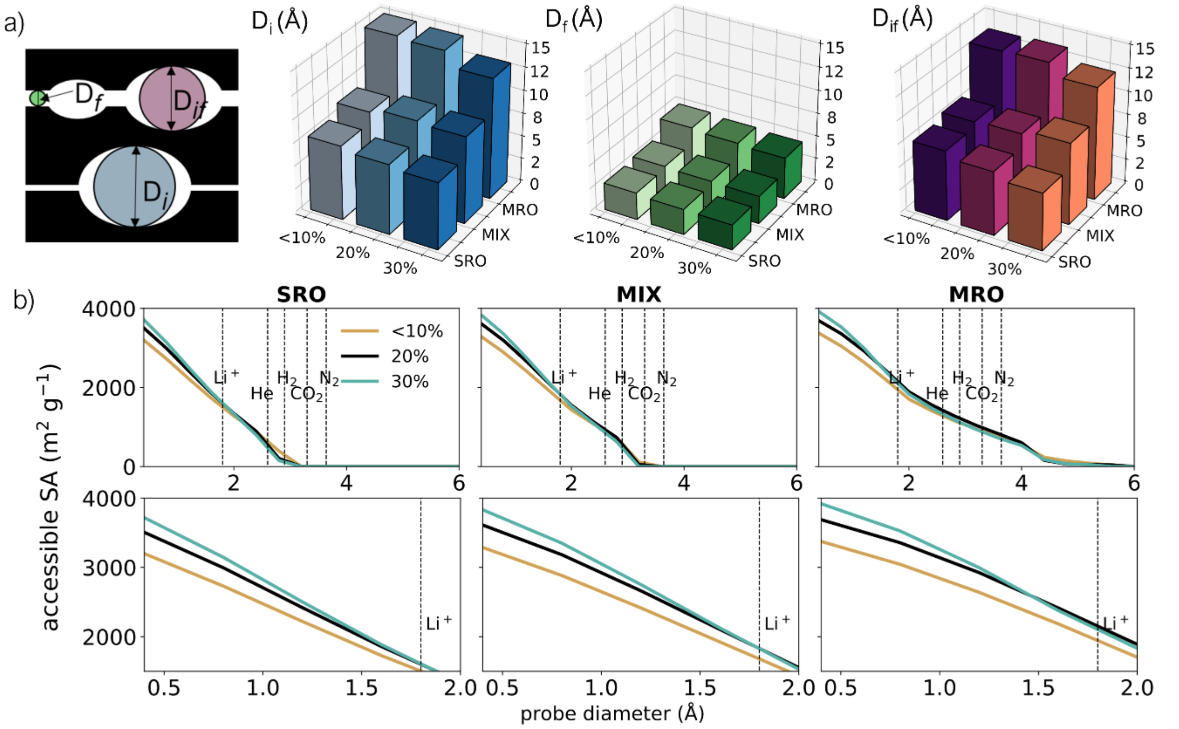

Figure S12 *a) Definition and average values of D_i (diameter of the largest sphere that can be included in the model), D_f (diameter of the largest sphere that can percolate through the model) and D_{if} (diameter of the largest sphere accessible along the path of D_f) for the nine studied phases. b) (top) Plot of accessible surface area (ASA) as a function of the probe diameter used to calculate it, for systems at the same level of order (SRO on the left, MIX in the center and MRO on the right) and different level of defects. The kinetic diameters of some common guest atoms and molecules are reported as comparison: if the dotted line crosses the ASA line, the model is porous to that guest. When considering accessibility for the guests that are slightly bigger than the D_f value of the models it is worth remembering that a certain degree of flexibility (not taken into account in our porosity calculation) could make the structure accessible to them. (bottom) Zoom-in of the plot on top between 1500 and 4000 m^2 g^{-1} (y-azis) and 0.40 and 2.0 Å (x-axis), which highlights the highest values of ASA in more defective structures for probes with diameter below 2.0 Å.*

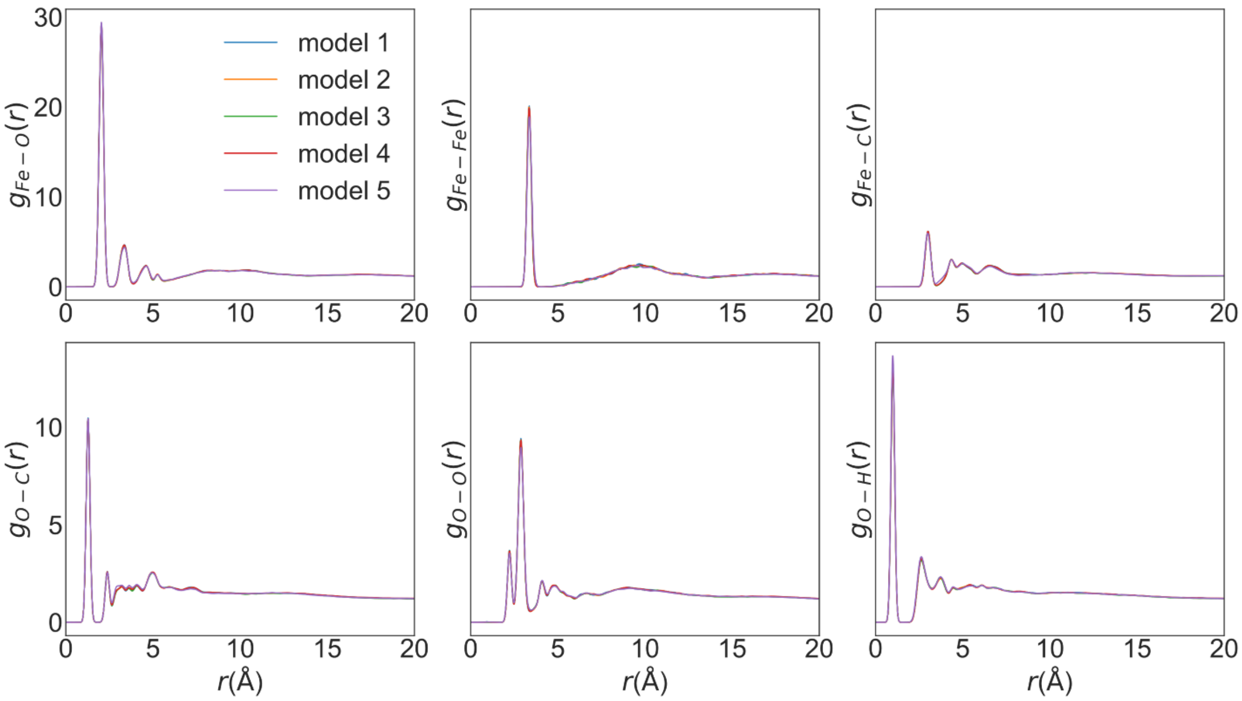

Figure S13 *Main partial PDFs (Fe-O, Fe-Fe, Fe-C, O-C, O-O, O-H) plotted for all the five models of the SRO <10% phase. Deviations between different models are very small.*

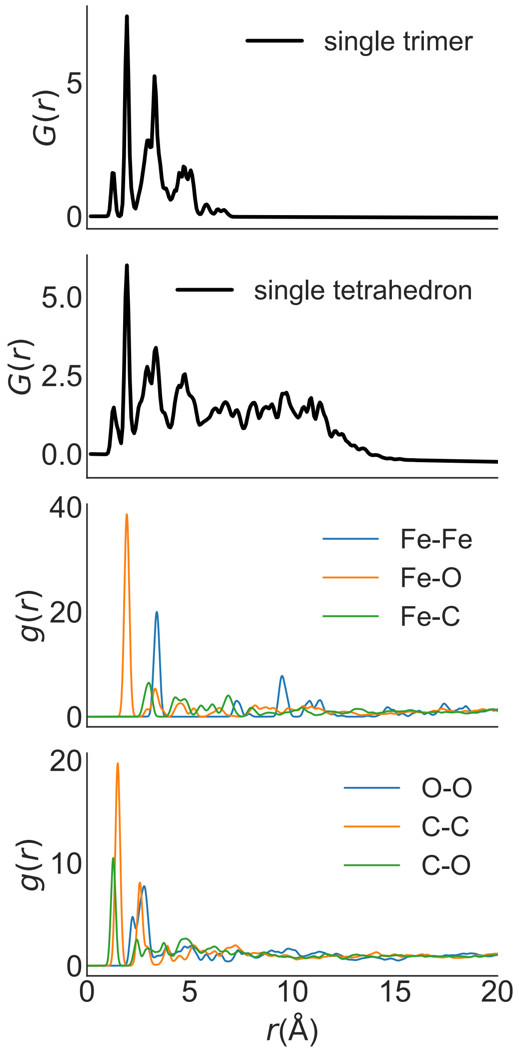

Figure S14 *(bottom plots) Partial PDFs calculated from the crystalline MIL-100(Fe), (first plot from above) PDFs of a single trimer and (second plot from above) a single tetrahedron. The Fe-O, C-O and O-O peaks are the dominating peaks.*

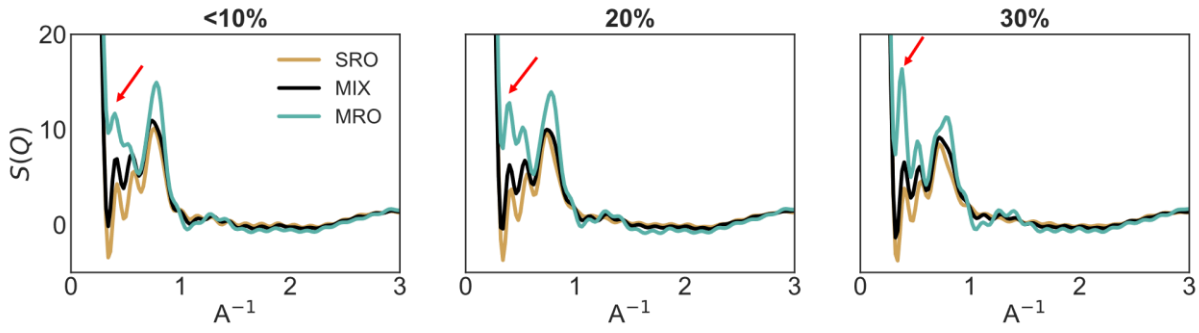

Figure S15 *Low Q region of the calculated structure factor S(Q) for the nine phases. Sharp peaks in this region for MRO models arise from oscillation between low-density and high-density areas within the model. The presence of the tetrahedra accentuates this density fluctuation and results in higher intensity peaks at low Q values (the feature is highlighted by the red arrow at 0.38* $Å^{-1}$ *and has a real-space period that is largely consistent with the size of the tetrahedral units). When this is Fourier transformed to obtain the PDF, it gives a broad oscillation and can contribute to the higher intensity peaks at r > 5 Å in MRO structures with higher defect content.*

Table S10 *Statistical variance (%) captured by each component of the PCA applied to a series of 3 PDFs. The distortion component (PC2) generally accounts for around 1 % of the statistical variance in the dataset.*

series	label	component	Percentage of variance	Cumulative variance
SRO 30%, MIX 30%, MRO 30%	30%	PC1	98.67%	98.67%
		PC2 (distortion)	1.24%	99.91%
		PC3	0.09%	100.00%
SRO 20%, MIX 20%, MRO 20%	20%	PC1	99.10%	99.10%
		PC2 (distortion)	0.81%	99.91%
		PC3	0.09%	100.00%
SRO <10%, MIX <10%, MRO <10%	<10%	PC1	99.43%	99.43%
		PC2 (distortion)	0.42%	99.85%
		PC3	0.15%	100.00%
SRO <10%, SRO 20%, SRO 30%	SRO	PC1	99.03%	99.03%
		PC2 (distortion)	0.87%	99.90%
		PC3	0.10%	100.00%
MIX <10%, MIX 20%, MRO 30%	MIX	PC1	99.04%	99.04%
		PC2 (distortion)	0.87%	99.91%
		PC3	0.09%	100.00%
MRO <10%, MRO 20%, MRO 30%	MRO	PC1	98.87%	98.87%
		PC2 (distortion)	1.03%	99.90%
		PC3	0.10%	100.00%
MRO <10%, MIX 20%, SRO 30%	diagonal	PC1	98.88%	98.88%
		PC2 (distortion)	0.99%	99.88%
		PC3	0.12%	100.00%

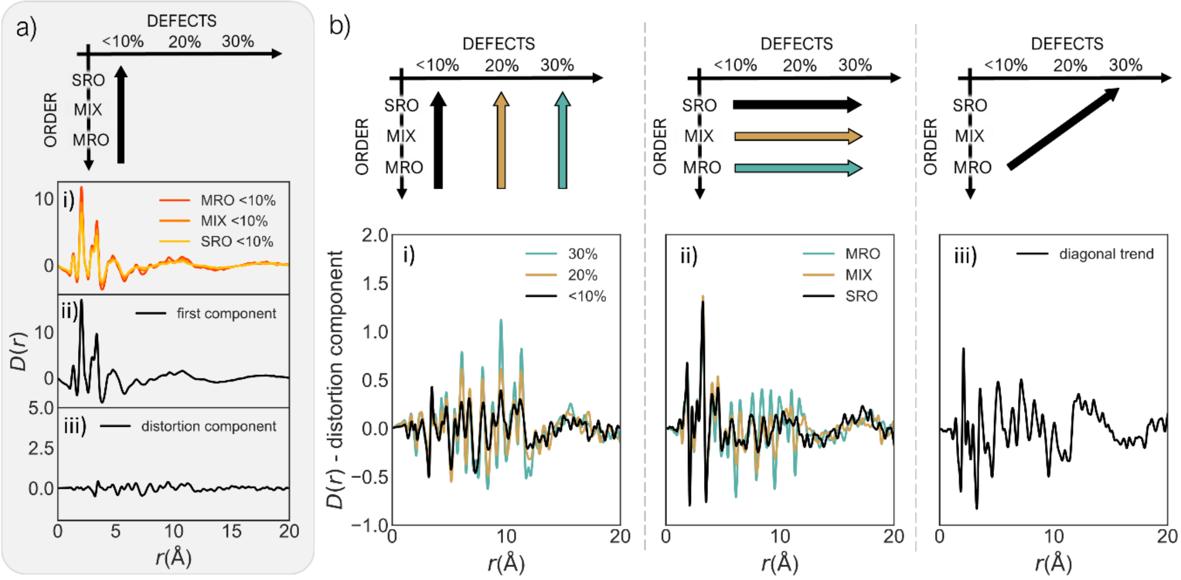

Figure S16 *a) Example of PCA applied to the trend shown in the phase diagram on top [SRO <10% - MIX <10% - MRO <10%]. The input data (i) is used to obtain the corresponding outputs (ii and iii), the second of which (iii) is the distortion component. b) Distortion components from the PCA analysis of PDFs for the 7 trends studied: i) the three trends in which the level of defects is kept constant, and the level of order changed (labelled <10%, 20%, 30%, according to the level of defects), ii) the three trends at constant level of order and varying level of defects (labelled MRO, MIX, SRO, based on the level of order) and iii) the diagonal trend that changes level of order and defects simultaneously on the right. The arrows across the phase diagram above each plot indicate the three phases used as input for each plotted output.*

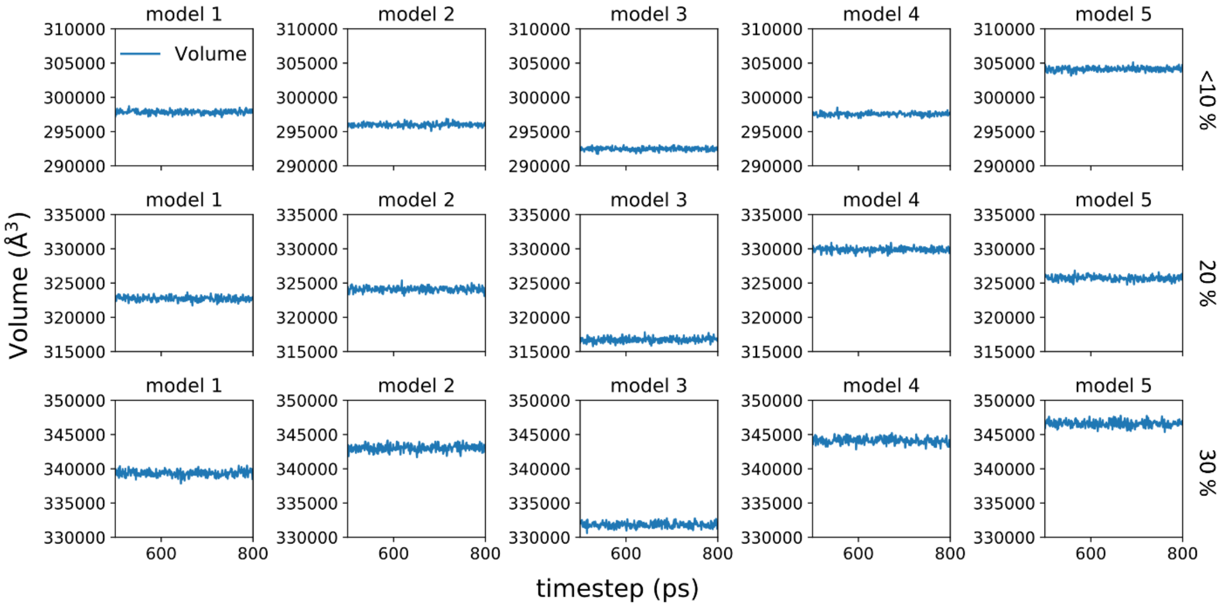

Figure S17 *Volume of SRO models (different models from left-to-right, change in degree of defects from top-to-bottom) over the final 300 ps of the annealing process.*

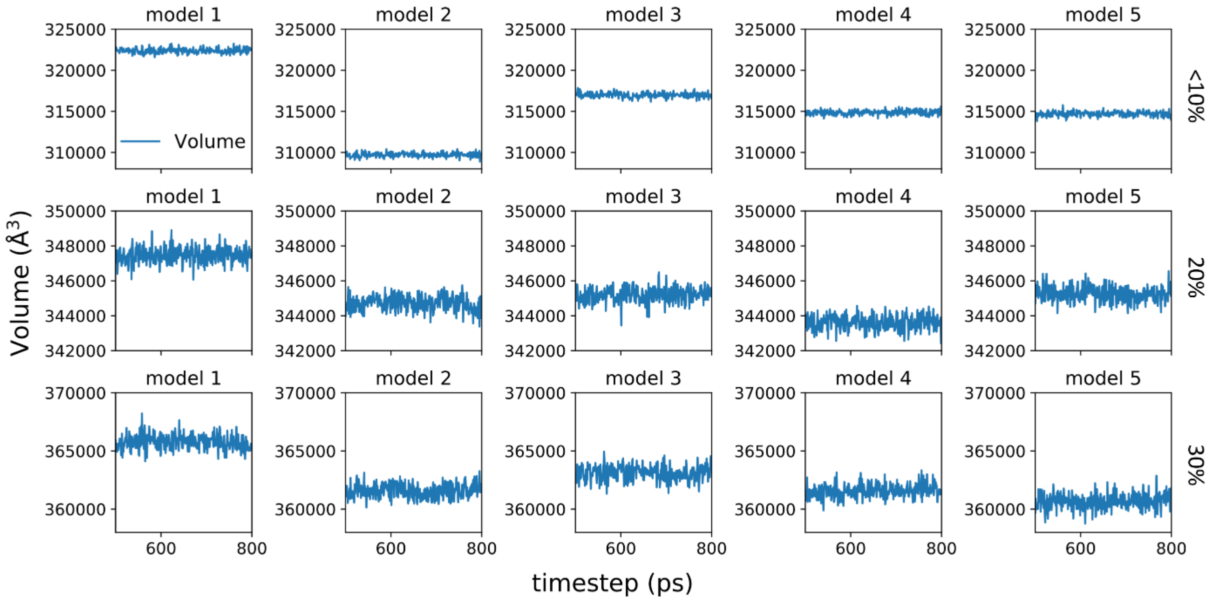

Figure S18 *Volume of MIX models (different models from left-to-right, change in degree of defects from top-to-bottom) over the final 300 ps of the annealing process.*

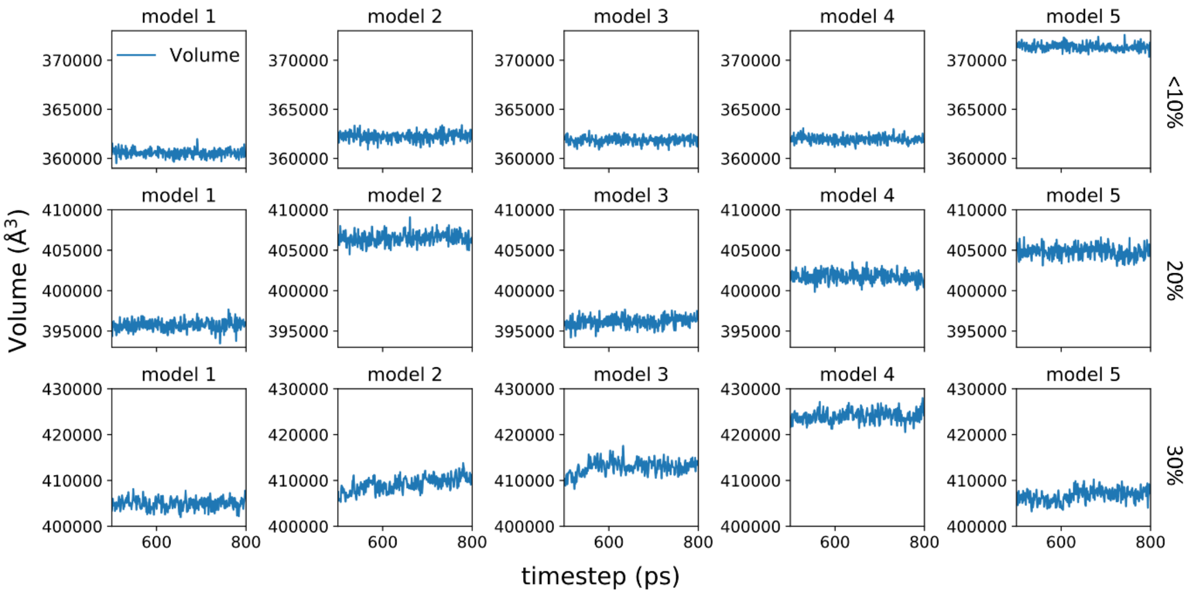

Figure S19 *Volume of MRO models (different models from left-to-right, change in degree of defects from top-to-bottom) over the final 300 ps of the annealing process.*

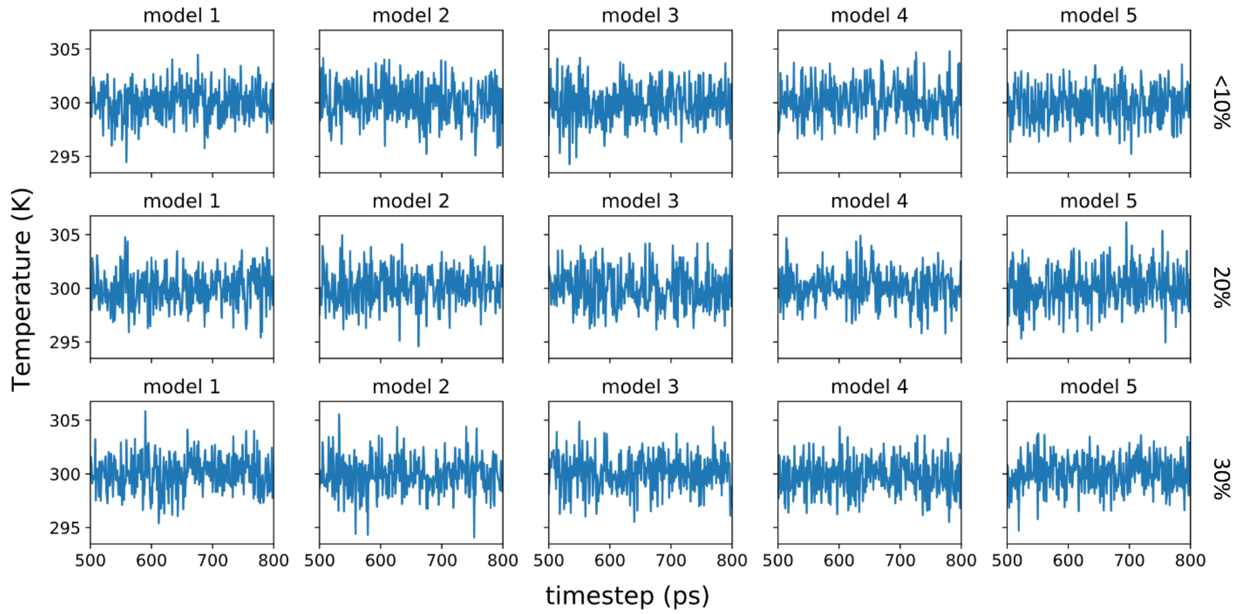

Figure S20 *Temperature of SRO models (different models from left-to-right, change in degree of defects from top-to-bottom) over the final 300 ps of the annealing process.*

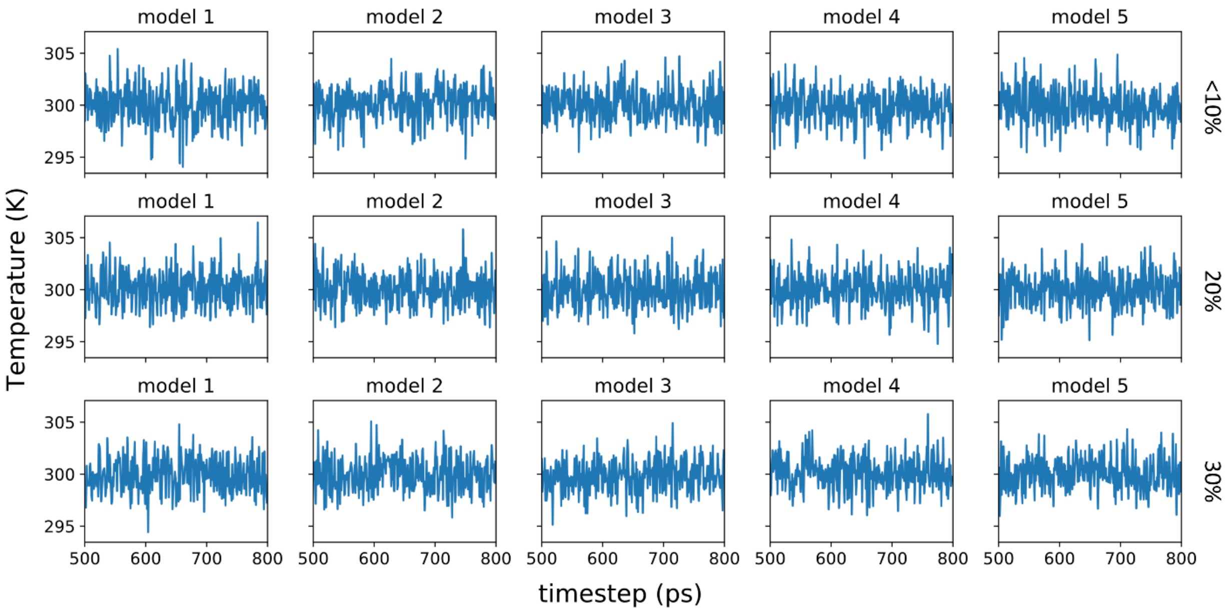

Figure S21 *Temperature of MIX models (different models from left-to-right, change in degree of defects from top-to-bottom) over the final 300 ps of the annealing process.*

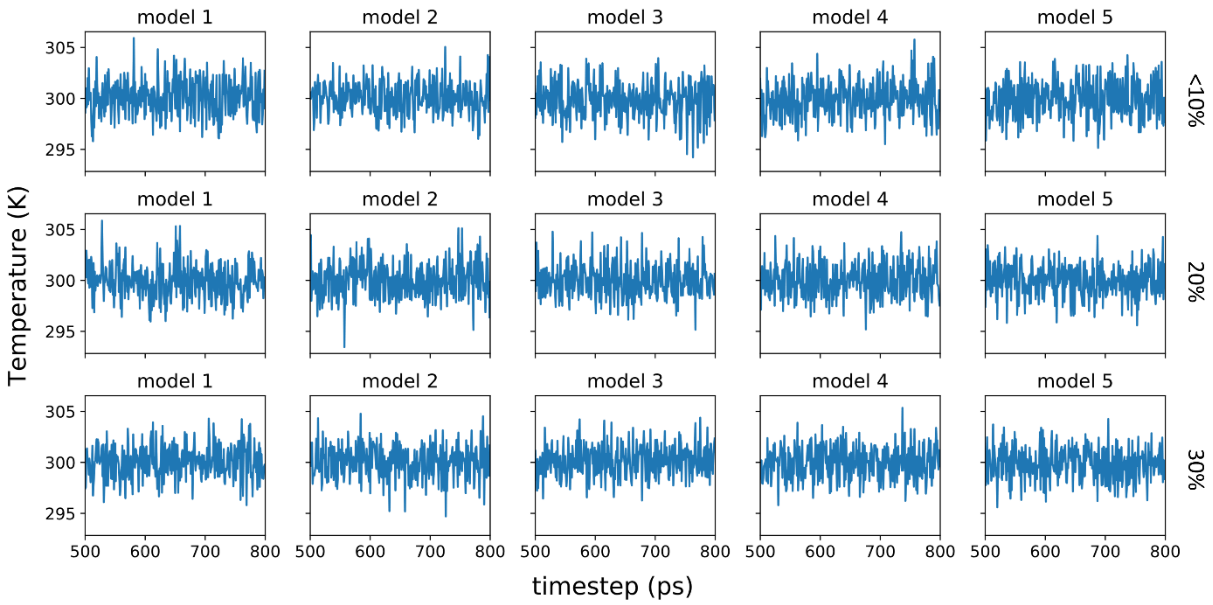

Figure S22 *Temperature of MRO models (different models from left-to-right, change in degree of defects from top-to-bottom) over the final 300 ps of the annealing process.*

References

1. Rappe, A. K., Casewit, C. J., Colwell, K. S., Goddard, W. A. & Skiff, W. M. UFF, a full periodic table force field for molecular mechanics and molecular dynamics simulations. *J. Am. Chem. Soc.* **114**, 10024–10035 (1992).
2. Addicoat, M. A., Vankova, N., Akter, I. F. & Heine, T. Extension of the Universal Force Field to Metal−Organic Frameworks. *J. Chem. Theory Comput.* **10**, 880−891 (2014).
3. Coupry, D. E., Addicoat, M. A. & Heine, T. Extension of the Universal Force Field for Metal−Organic Frameworks. *J. Chem. Theory Comput.* **12**, 5215−5225 (2016).
4. Boyd, P. G., Moosavi, S. M., Witman, M. & Smit, B. Force-Field Prediction of Materials Properties in Metal-Organic Frameworks. *J. Phys. Chem. Lett.* **8**, 357–363 (2017).
5. Thompson, A. P. *et al.* LAMMPS - a flexible simulation tool for particle-based materials modeling at the atomic, meso, and continuum scales. *Comput. Phys. Commun.* **271**, 108171 (2022).
6. Sapnik, A. F. *et al.* Mixed hierarchical local structure in a disordered metal-organic framework. *Nat. Commun.* **12**, 2062 (2021).
7. Frisch, M. J. *et al.* Gaussian16. (2016).
8. Stephens, P. J., Devlin, F. J., Chabalowski, C. F. & Frisch, M. J. Ab Initio Calculation of Vibrational Absorption and Circular Dichroism Spectra Using Density Functional Force Fields. *J. Phys. Chem.* **98**, 11623–11627 (1994).
9. Grimme, S., Ehrlich, S. & Goerigk, L. Effect of the Damping Function in Dispersion Corrected Density Functional Theory. *J. Comput. Chem.* **32**, 1456–1465 (2011).
10. Gale, J. D. & Rohl, A. L. The General Utility Lattice Program (GULP). *Mol. Simul.* **29**, 291–341 (2003).
11. Horcajada, P. *et al.* Synthesis and catalytic properties of MIL-100(Fe), an iron(III) carboxylate with large pores. *Chem. Commun.* **27**, 2820–2822 (2007).
12. Chung, Y. G. *et al.* Computation-Ready, Experimental Metal−Organic Frameworks: A Tool To Enable High-Throughput Screening of Nanoporous Crystals. *Chem. Mater.* **26**, 6185−6192 (2014).
13. Chung, Y. G. *et al.* Advances, Updates, and Analytics for the Computation-Ready, Experimental Metal−Organic Framework Database: CoRE MOF 2019. *J. Chem. Eng. Data* **64**, 5985−5998 (2019).
14. Accelrys Software Inc. Material Studio. (2007).
15. Abbott, L. J., Hart, K. E. & Colina, C. M. Polymatic: A generalized simulated polymerization algorithm for amorphous polymers. *Theor. Chem. Acc.* **132**, 1334 (2013).
16. Larsen, G. S., Lin, P., Hart, K. E. & Colina, C. M. Molecular Simulations of PIM-1-like Polymers of Intrinsic Microporosity. *Macromolecules* **44**, 6944–6951 (2011).
17. Stukowski, A. Visualization and analysis of atomistic simulation data with OVITO–the Open Visualization Tool. *Model. Simul. Mater. Sci. Eng.* **18**, 015012 (2010).
18. Zimmermann, N. E. R. & Jain, A. Local structure order parameters and site fingerprints for quantification of coordination environment and crystal structure similarity. *RSC Adv.* **10**, 6063–6081 (2020).
19. Ping Ong, S. *et al.* Python Materials Genomics (pymatgen): A robust, open-source python library for materials analysis. *Comput. Mater. Sci.* **68**, 314–319 (2013).
20. Michaud-Agrawal, N., Denning, E. J., Woolf, T. B. & Beckstein, O. MDAnalysis: A toolkit for the analysis of molecular dynamics simulations. *J. Comput. Chem.* **32**, 2319–2327 (2011).
21. Gowers, R. *et al.* MDAnalysis: A Python Package for the Rapid Analysis of Molecular Dynamics Simulations. in *Proceedings of the 15th Python in Science Conference* 98–105 (2016).
22. Hagberg, A. A., Schult, D. A. & Swart, P. J. Exploring Network Structure, Dynamics, and Function using NetworkX. in *Proceedings of the 7th Python in Science conference* 11–15 (2008).

23. Willems, T. F., Rycroft, C. H., Kazi, M., Meza, J. C. & Haranczyk, M. Algorithms and tools for high-throughput geometry-based analysis of crystalline porous materials. *Microporous Mesoporous Mater.* **149**, 134–141 (2012).
24. Robeson, L. M. Correlation of separation factor versus permeability for polymeric membranes. *J. Memb. Sci.* **62**, 165–185 (1991).
25. Tucker, M. G., Keen, D. A., Dove, M. T., Goodwin, A. L. & Hui, Q. RMCProfile: reverse Monte Carlo for polycrystalline materials. *J. Phys. Condens. Matter* **19**, 335218 (2007).
26. Keen, D. A. A comparison of various commonly used correlation functions for describing total scattering. *J. Appl. Cryst.* **34**, 172–177 (2001).
27. Sapnik, A. F. *et al.* Multivariate analysis of disorder in metal-organic frameworks. *Nat. Commun.* **13**, 2173 (2022).
